# Fluorescence lifetime FRET assay for live-cell high-throughput screening of the cardiac SERCA pump yields multiple classes of small-molecule allosteric modulators

**DOI:** 10.1038/s41598-023-37704-x

**Published:** 2023-07-01

**Authors:** Osha Roopnarine, Samantha L. Yuen, Andrew R. Thompson, Lauren N. Roelike, Robyn T. Rebbeck, Philip A. Bidwell, Courtney C. Aldrich, Razvan L. Cornea, David D. Thomas

**Affiliations:** 1 Department of Biochemistry, Molecular Biology and Biophysics, University of Minnesota, Minneapolis, MN USA; 2 Department of Medicine, Cardiovascular Division, University of Minnesota, Minneapolis, MN 55455 USA; 3 Department of Medicinal Chemistry, University of Minnesota, Minneapolis, MN 55455 USA

**Keywords:** High-throughput screening, Heart failure, Drug discovery, Drug screening, Cheminformatics, Fluorescence resonance energy transfer, Fluorescence spectroscopy, Calcium channels, Membrane proteins

## Abstract

We have used FRET-based biosensors in live cells, in a robust high-throughput screening (HTS) platform, to identify small-molecules that alter the structure and activity of the cardiac sarco/endoplasmic reticulum calcium ATPase (SERCA2a). Our primary aim is to discover drug-like small-molecule activators that improve SERCA’s function for the treatment of heart failure. We have previously demonstrated the use of an intramolecular FRET biosensor, based on human SERCA2a, by screening two different small validation libraries using novel microplate readers that detect the fluorescence lifetime or emission spectrum with high speed, precision, and resolution. Here we report results from FRET-HTS of 50,000 compounds using the same biosensor, with hit compounds functionally evaluated using assays for Ca^2+^-ATPase activity and Ca^2+^-transport. We focused on 18 hit compounds, from which we identified eight structurally unique scaffolds and four scaffold classes as SERCA modulators, approximately half of which are activators and half are inhibitors. Five of these compounds were identified as promising SERCA activators, one of which activates Ca^2+^-transport even more than Ca^2+^-ATPase activity thus improving SERCA efficiency. While both activators and inhibitors have therapeutic potential, the activators establish the basis for future testing in heart disease models and lead development, toward pharmaceutical therapy for heart failure.

## Introduction

Sarco/endoplasmic reticulum calcium ATPase (SERCA), integral to the sarcoplasmic reticulum (SR, muscle) or endoplasmic reticulum (ER, non-muscle) membrane in most mammalian cells, uses Ca^2+^-dependent hydrolysis of ATP to fuel active transport (uptake) of cytosolic Ca^2+^ into the SR or ER. The activity of SERCA1a (skeletal isoform) or SERCA2a (cardiac isoform) is essential for muscle relaxation (diastole), restoring SR Ca^2+^ following its release via Ca^2+^ channels (ryanodine receptors, RyR) for muscle contraction (systole). Decreased SERCA activity and excessive RyR leak results in failure to maintain the high gradient of [Ca^2+^] between the cytoplasm (sub-μM) and the SR (mM) during diastole and are associated with heart failure (HF) in humans and animals^1^. Decreased SERCA activity is related to multiple factors, including reduced SERCA gene expression, increased post-translational modifications, and altered interaction with regulatory proteins^1^. Overall, decreased SERCA activity and increased Ca^2+^-leak can lead to a pathophysiological state of the cardiac myocyte^2^ (HF, cardiac hypertrophy, diabetic hypertrophy), skeletal myofiber (Brody’s disease and myotonic dystrophy)^3^, or non-muscle cells (Darier’s disease, diabetes, Alzheimer’s disease)^4^. Altered SERCA interactions with regulatory proteins (regulins), e.g., phospholamban (PLB), have been linked to HF^5^. Of the seven known regulins^6^, the dwarf open reading frame (DWORF) peptide is the only one known to activate SERCA, both by direct activation^7,8^ and by competing with PLB binding^9,10^, preventing HF in a mouse model of dilated cardiomyopathy^11^.

Current therapeutic measures for HF include beta-blockers, angiotensin-converting enzyme (ACE) inhibitors, and angiotensin-receptor blockers (ARB). However, these do not directly target proteins responsible for dysfunctional Ca^2+^ cycling. Discovery of small molecules that target specific transporters, and their interaction with modulatory proteins, is needed to exert improved control of Ca^2+^ homeostasis for positive therapeutic outcomes. Here, we seek primarily SERCA2a activators to alleviate HF and related arrhythmias, a goal supported by numerous reports^12,13^, although some controversy remains^14^. Compounds that uncouple ATPase and transport activities of SERCA are of interest as enhancers of metabolism or thermogenesis, to reduce obesity or hypothermia^15,16^. SERCA inhibitors are proposed for treatment of cancer or malaria^17,18^.

**Figure 1:**
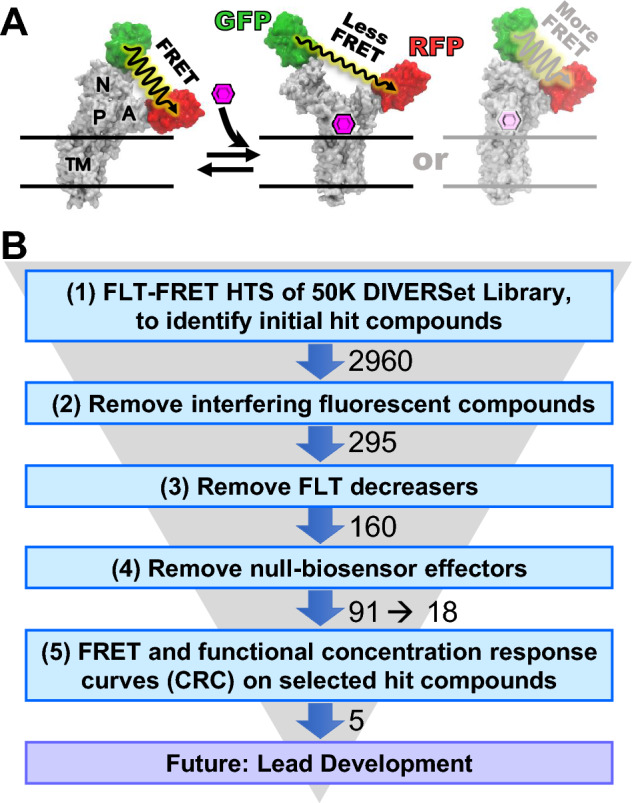
Strategy of this study. (**A**) FRET biosensor, human two-color SERCA2a (2CS), showing SERCA domains: nucleotide binding (N), phosphorylation (P), actuator (A) and transmembrane (TM). GFP is fused to N, RFP to A. ΔFRET (change in fluorescence resonance energy transfer), calculated from ΔFLT (change in fluorescence lifetime), is used to detect SERCA structural changes induced by compounds. The representative structural states show domain movements based on crystal structures from different nucleotide-bound SERCAs that indicate possible extents of FRET between GFP and RFP in the absence and presence of a compound (small molecule) (⎔): FRET (E2 + Tg (1IWO)), Less FRET (E1 + Ca^2+^, 1SU4), and More FRET (E1 + Ca^2+^ + ADP + AlF, 2ZBD)^19^. Here we focus on hits that decrease FRET (increase FLT). (**B**) Screening funnel describing the 5-step process in this study, involving measurements of FLT-FRET and SERCA function, with SERCA in live mammalian cells (HEK293) and in isolated pig cardiac SR membranes, respectively: (1) FLT changes caused by test compounds were measured using the SERCA-specific FRET biosensor 2CS (two-color SERCA), to identify 2960 initial hit compounds. False hits were ruled out as compounds that (2) are fluorescent or affect the donor directly, decreasing the hit compounds to 295, (3) decrease FLT (increase FRET), reducing the hit compounds to 160, and (4) affect FRET in a null-biosensor, in which donor and acceptor are separated by a non-functional flexible peptide, reducing the hit compounds to 91, of which 18 were selected for step 5 (see Results under “Removal of null-biosensor effectors”). (5) Compound concentration-dependence of FRET and SERCA function was measured to further prioritize hit compounds for future lead development. Experimental details are provided in Methods.

SERCA2a is a large transmembrane protein, with the phosphorylation (P) and nucleotide-binding (N) domains forming the catalytic site, influenced by the actuator (A) domain (Fig. 1A). Large (5–10 nm) relative movements of these domains, detected in living cells by an intramolecular FRET biosensor (Fig. 1A)^20,21^, are coupled to Ca^2+^ transport. The interaction of small molecules with SERCA can induce structural changes, detectable by this biosensor, that correlate with function, making this a powerful tool for high throughput screening (HTS) discovery of SERCA-binding compounds^20,22^.

In previous early-stage drug discovery campaigns, we focused on the SERCA regulator, PLB, via *inter*molecular FRET biosensor designs^23,24^. We have also validated *intra*molecular FRET biosensor constructs of SERCA^20,22,25^, to detect binding of compounds directly to SERCA. For this, we engineered a “two-color” SERCA (2CS, Fig. 1A) construct with eGFP and tagRFP fluorescent proteins fused to the cytoplasmic N- and A-domains of SERCA, to detect relative motions of these domains during the enzymatic cycle responsible for Ca^2+^-transport^22,25,26^. Measuring FRET within 2CS, stably expressed in a mammalian cell line (HEK293), we previously validated this biosensor using the NCC (727 compounds)^22^ and LOPAC (1280 compounds)^25^ libraries. The next logical step, in the present study, is to use 2CS in HTS of a 50,000-compound DIVERSet library, a diverse collection of drug-like small molecules that has yielded effective hit compounds in other drug discovery projects^27–29^. HTS was enabled by the FLT-PR (fluorescence lifetime plate reader), which scans a 1536-well plate with unprecedented precision and speed, determining FLT with ~ 0.3% CV (30 times better precision than conventional intensity detection) in 2.5 min^23,27,28^, making possible a high-precision 50,000-compound screen in 2 days. To remove false positives, a spectral unmixing plate reader (SUPR) was used to provide complementary spectral measurement of compound-induced FRET changes^26^.

Although other biosensors are under development (e.g., using orange and maroon fluorescent proteins)^22,23,26^, the GFP/RFP 2CS biosensor has been thoroughly validated for screening the DIVERSet compound library (DIVERSet-CL) using HEK293 cells. To validate selected hit compounds and prioritize those with lead potential, we acquired concentration response curves (CRCs) using FRET and functional assays (Ca^2+^-ATPase activity and Ca^2+^-uptake). We hypothesized that the combination of improved fluorescence technology and screening a larger library of compounds would yield a larger and more diverse collection of hit compounds that improve cardiac SERCA function, thus increasing the potential for discovering lead compounds for new heart failure therapeutics.

## Results

### FLT-FRET HTS of 50K DIVERSet-CL

**Figure 2:**
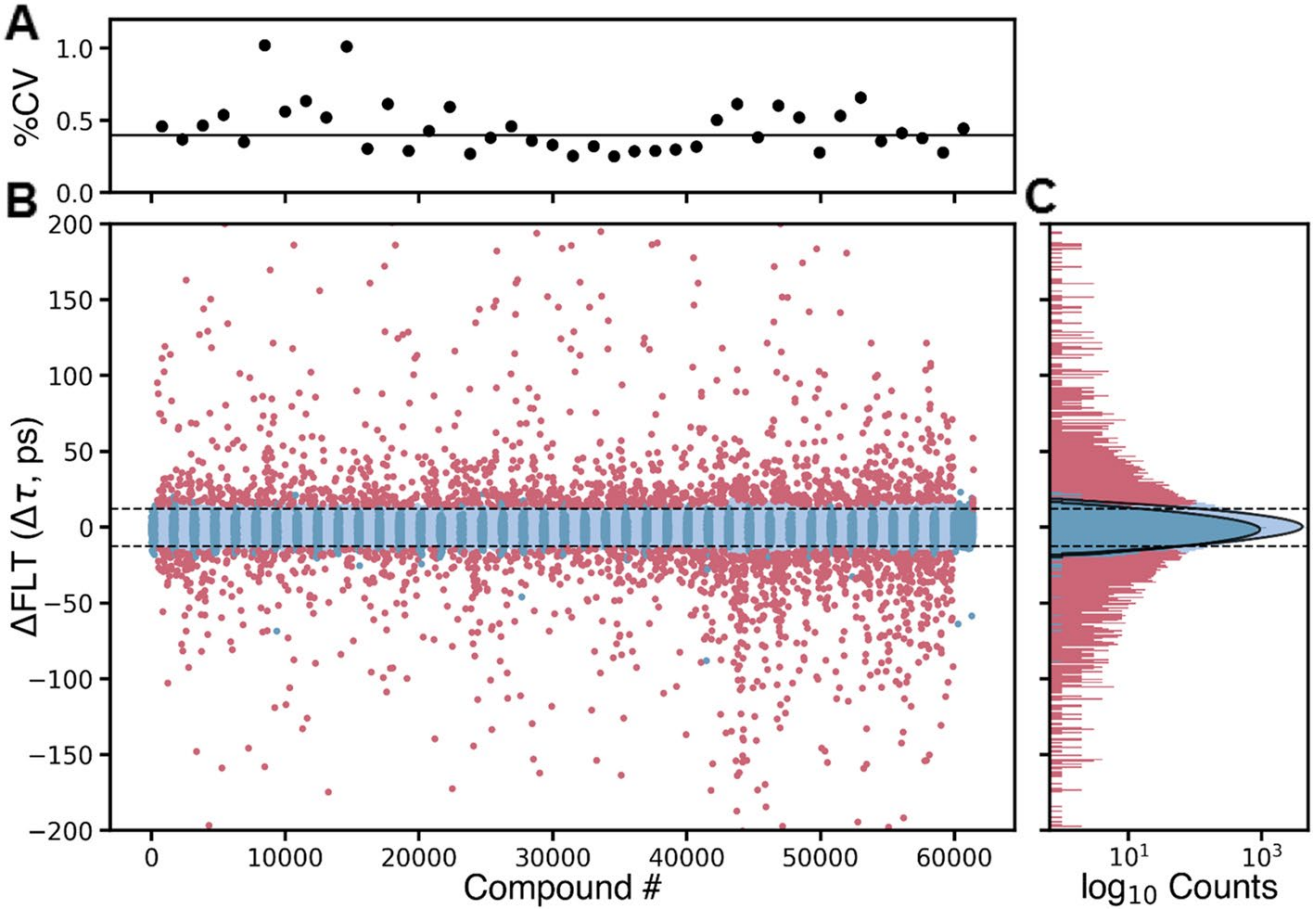
FLT HTS of the DIVERSet library of 50,000-compounds, using the approach described in Fig. 1A to identify initial hit compounds. (**A**) Screening precision was determined by computing %CV for each plate using DMSO control wells, with a median value of 0.4% across 40 plates. (**B**) The change in lifetime (Δτ) was computed to find potential hits (red) with hit threshold set at r*Z*-score =  ± 3, resulting in 2960 initial hits for triage with the SUPR instrument. DMSO controls (dark blue) and compounds not affecting 2CS (light blue) are grouped in the plot to illustrate plate boundaries. (**C**) The histogram of compounds not affecting 2CS (light blue, 1 ps bin width) shows normal distribution, similar to that of DMSO controls (no compound), as shown by a fit of the populations to Gaussian distributions. The horizontal lines in (**B,C**) illustrate the approximate cutoffs used, though actual cutoffs were determined on a plate-by-plate basis.

The 2CS FRET biosensor^20,22,25^ was incubated with compounds or DMSO (control) for FLT acquisition in the FLT-PR. FLT measurements had a median coefficient of variation (CV) of 0.4% across all plates (Fig. 2A). Plate-by-plate CV varied by < 1% (Fig. 2A).

Compounds that significantly altered the structure of 2CS were determined from their change in lifetime (Δτ) vs DMSO controls (2CS plus DMSO), and Δτ was compared to the normal statistical fluctuation of the biosensor by computing the robust (r)*Z*-score (see Methods). FLT changes induced by potential hit compounds (Fig. 2B, red) are distinct from the normal distributions of DMSO controls (Fig. 2C, dark blue) and of compounds not affecting SERCA2a (Fig. 2C, light blue). A hit threshold was set at r*Z*-score =  ± 3, resulting in 2960 initial hit compounds (Fig. 2B, step 1), which is ~ 10 × more initial hit compounds than we previously identified with an ATPase-based HTS assay^30^ using a similar threshold. Optically interfering compounds were removed (Fig. 1B, step 2) using a spectral unmixing plate reader to obtain spectra for 2CS that were analyzed relative to the donor-only 1CS sample^22^ (SUPR, see Methods). 295 compounds remained.

We cannot predict the direction of ΔFLT for activator vs. inhibitor. However, FLT increasers were preferred because: (a) more FLT decreasers were found to fail these tests^23,25,26,31^, (b) increasers offer greater reproducibility^23,25,26^, and (c) most previously identified SERCA modulators have been shown to be FLT increasers^20,22,25^. Therefore, we prioritized 160 FLT increasers (termed “hit compounds” (Fig. 1B, step 3) for retesting with a null-biosensor, to remove false positives.

### Removal of null-biosensor effectors

**Figure 3:**
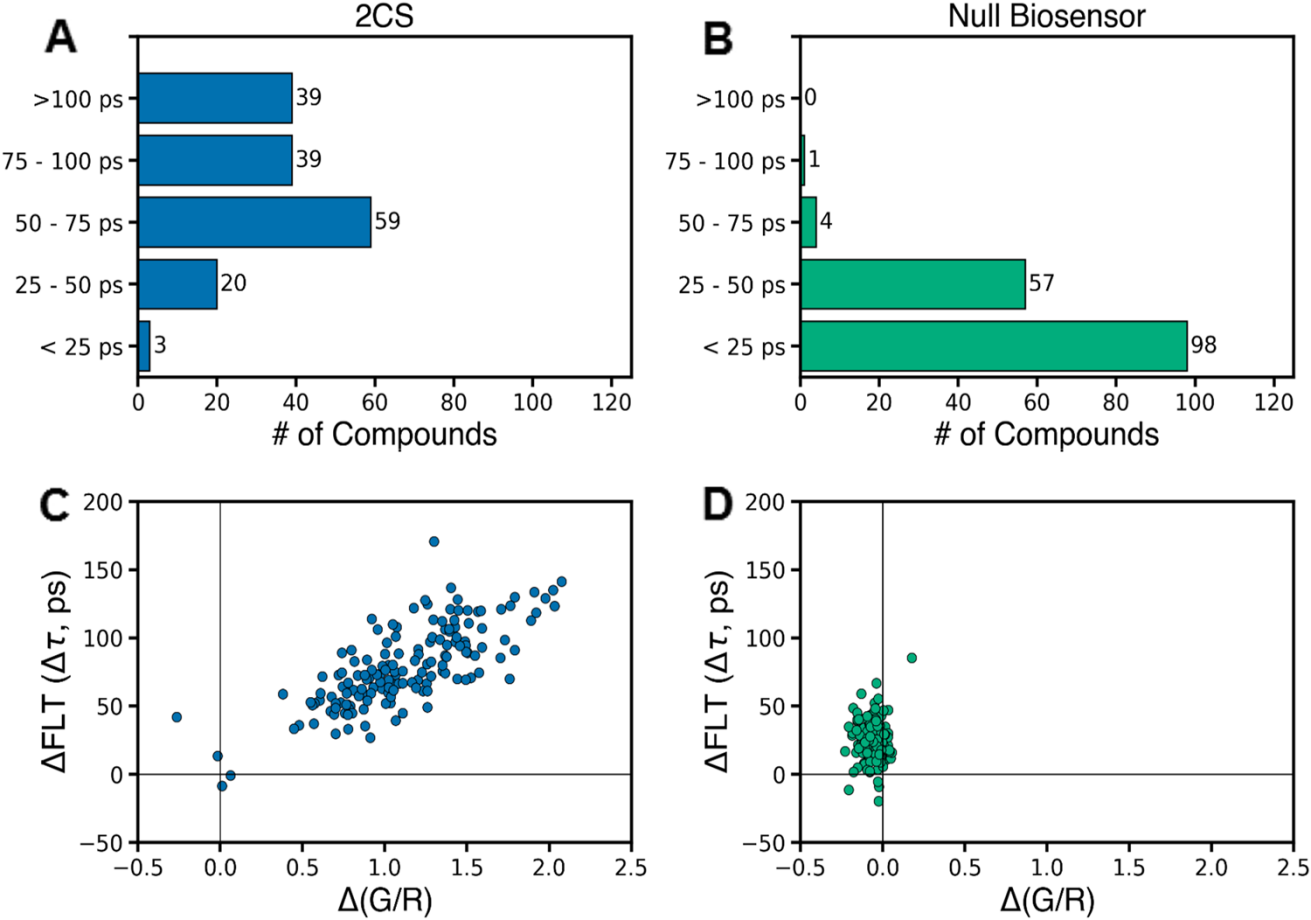
Removal of null-biosensor effectors (false positives). The 160 hit compounds that passed steps 1–3 of the screening funnel in Fig. 1B were then selected and dispensed into 1536-well plates containing 10 and 30 μM [compound] (n = 3 wells for each concentration), using the same compound stock solutions as in the HTS phase (step 1 in Fig. 1B). These repeat readings establish the reproducibility needed to select a set of hit compounds for re-purchase as solids, for subsequent studies discussed below. Data are shown from the 30 μM wells for 2CS (**A,C**) and null-biosensor (**B,D**). (**A**) Distribution of significant ΔFLT for the 2CS biosensor. (**B**) The 160 hit compounds were counter-screened using a null-biosensor. Only five compounds displayed Δτ > 50 ps, indicating that our method for eliminating fluorescent compounds removes nearly all false positives. These five compounds were also excluded from further consideration (Supplementary Fig. S1A). (**C**) and (**D**) Plots of ΔFLT vs. Δ(G/R) show excellent, reproducible correlation between the two measurements for the 2CS biosensor (**C**), but distinct from those observed for the null-biosensor (**D**), indicating that the hit compounds induce a structural change in the 2CS biosensor.

160 hit compounds were retested (Fig. 1B, step 4) using 2CS (Fig. 3A and C; Supplementary Fig. S1) and a null-biosensor (Fig. 3B and D), GFP and RFP connected by a 32-residue unstructured flexible linker peptide (G32R)^25^, to rule out compounds that directly bind to the fluorescent proteins and alter FLT. ΔFLT (Δτ) from the FLT-PR and Δ(G/R) (change in the ratio of mole fractions of donor [green, G] and acceptor [red, R] in the emission spectrum as determined from a linear combination of component spectra of donor-only and acceptor-only (1CS) samples^22^) from the SUPR were determined for each compound. As these are two complementary measures of FRET (FRET decreases both), a strong correlation was observed in 2CS for compounds that induced a structural change much greater than observed in the null-biosensor (Fig. 3C and D) (see Methods under “FRET-HTS instrumentation and data analysis”).

Hit compounds that produced Δτ ≥ 70 ps (91 compounds, Fig. 1B, step 4; Fig. 3A, Supplementary Fig. S1A), but excluding any that exceeded a 50 ps response in the null-biosensor, were targeted for further functional testing. After determining compound availability for repurchase, we selected 18 compounds with a representative range of ΔFLT (Supplementary Fig. S1B) for CRC testing. None of these compounds were in the PAINS (Pan-Assay INterference compoundS) category^32^, nor were they redox agents or metal chelators.

### Validation of hit compounds using FRET CRC

**Figure 4:**
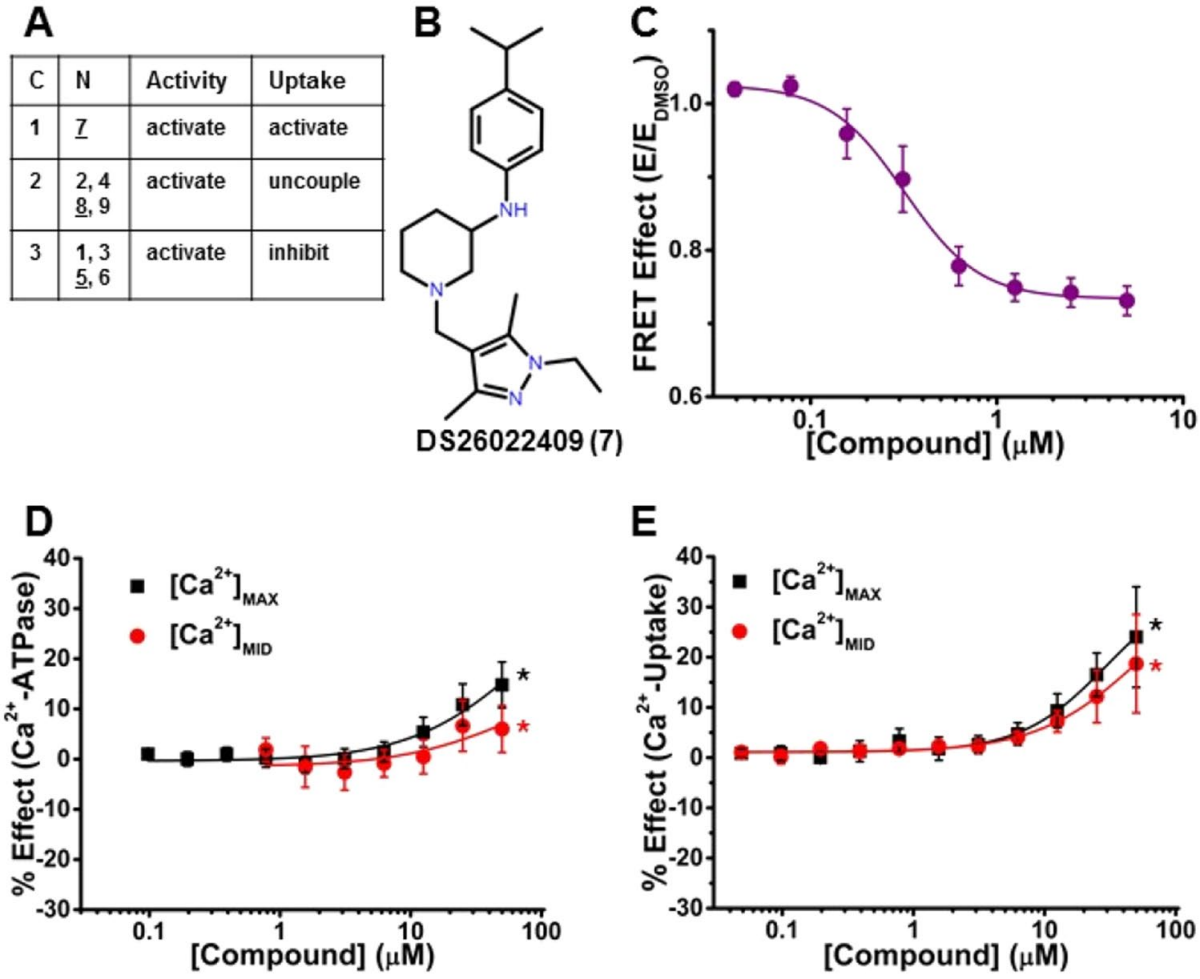
A representative activator enhances both Ca^2+^-ATPase activity and Ca^2+^-uptake at [Ca^2+^]_MAX_ (pCa = 5.4) and [Ca^2+^]_MID_ (pCa = 6.2). (**A**) Table showing the three categories (**C**) of activators (N, Compounds 1–9) and their effects on the Ca^2+^ATPase activity and Ca^2+^-uptake to activate, uncouple, or inhibit function. Underlined compounds are represented in Figs. 4, 5, and 6. (**B**) Chemical structure of DS26022409 (Compound 7). (**C**) CRC of normalized FRET E in live HEK cells shows decreasing FRET in response to increasing [compound]. (**D**) CRC of Ca^2+^-ATPase activity of SERCA2a in pCSR vesicles show activation at [Ca^2+^]_MAX_ (black) and at [Ca^2+^]_MID_ (red). (**E**) CRC of Ca^2+^-uptake shows activation at both [Ca^2+^]_MAX_ and [Ca^2+^]_MID_ (black and red, respectively). ΔF_MAX_, ΔF_MID_, C_10_, and EC_50_ are defined and reported in Table 1 for panels C, D, and E. Data are presented as mean ± SEM, n = 3, *p < 0.05.

**Figure 5:**
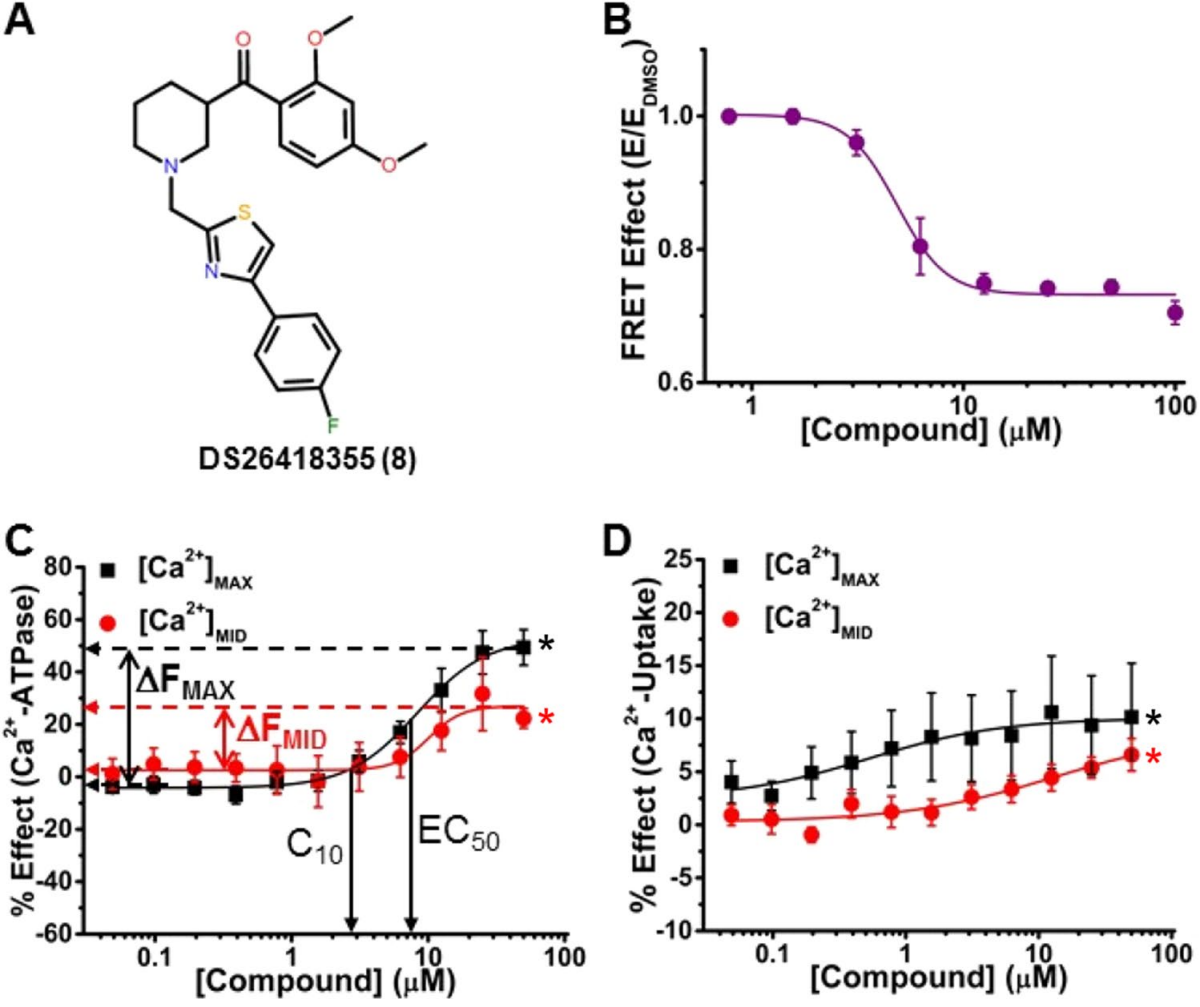
A representative activator that decreases FRET and uncouples Ca^2+^-ATPase activity from Ca^2+^-uptake activity. (**A**) Chemical structure of DS26418355 (Compound 8). (B) CRC of normalized FRET E in 2CS biosensor in live HEK cells shows decreasing FRET response with increasing [compound]. (**C**) CRC shows Ca^2+^-ATPase activation in pCSR vesicles at both [Ca^2+^]_MAX_ (black) and [Ca^2+^]_MID_ (red). (**D**) Activation was less for Ca^2+^-uptake of SERCA2a in pCSR vesicles. ΔF_MAX_, ΔF_MID_, C_10_, and EC_50_ are defined and reported in Table 1 for panels B, C, and D. Data is presented as mean ± SEM, n = 3, *p < 0.05.

**Figure 6:**
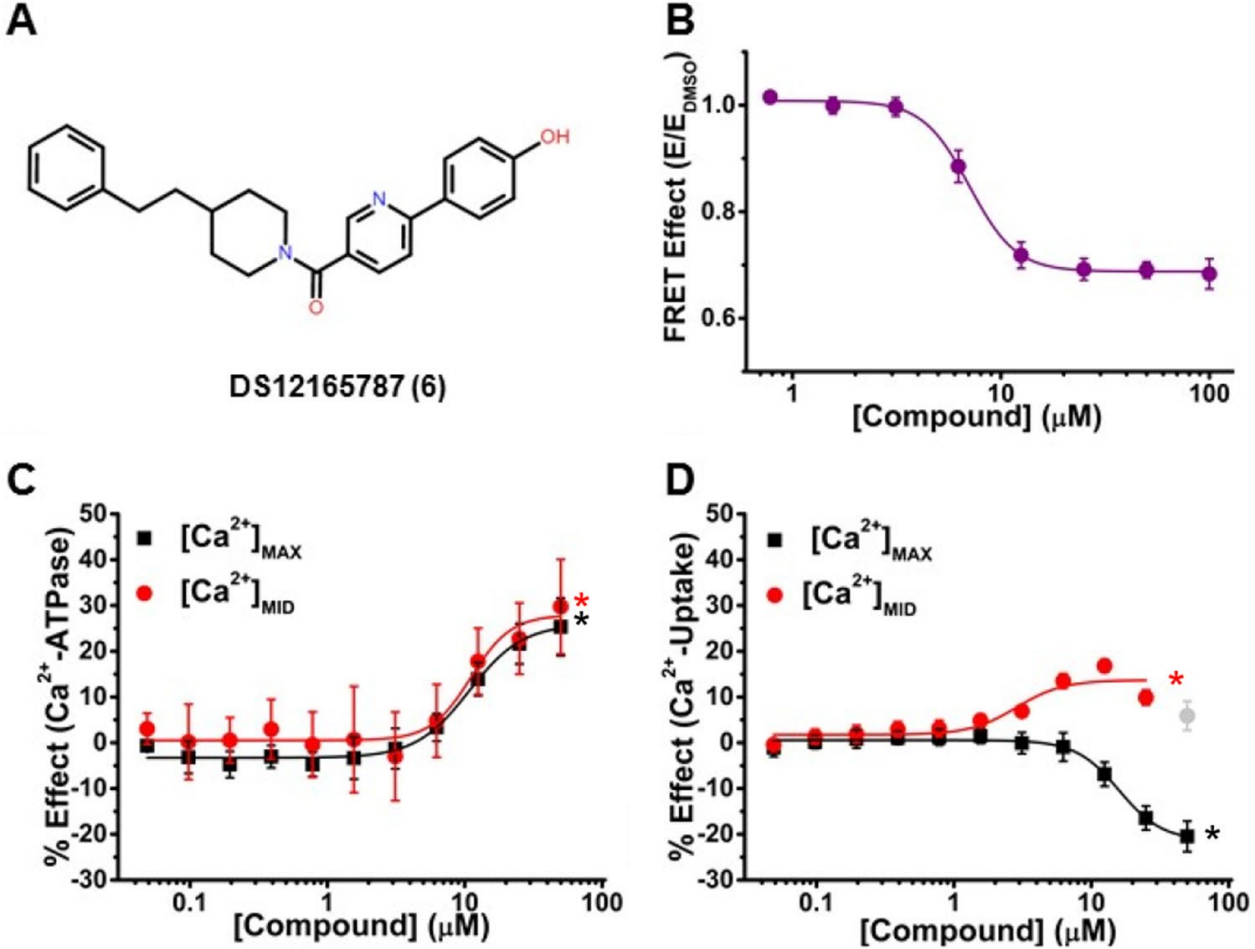
A representative activator that decreases FRET, increases Ca^2+^-ATPase activity, and has divergent effects (inhibitory) on Ca^2+^-uptake activity. (A) Chemical structure of Compound DS12165787 (Compound 6). (B) CRC of normalized FRET E shows decreased FRET in live HEK cells with a half maximal effect (EC_50_) at 7.1 ± 0.2 μM. (C) CRC shows Ca^2+^-ATPase activity increase of SERCA2a in pCSR vesicles at [Ca^2+^]_MAX_ (black, pCa 5.4) and [Ca^2+^]_MID_ (red, pCa 6.2) (D) CRC of Ca^2+^-uptake of SERCA2a in pCSR vesicles, showing inhibition for [Ca^2+^]_MAX_ (black) and activation for [Ca^2+^]_MID_ (red). The grey data point was omitted from fitting, to account for hormesis^40^. ΔF_MAX_, ΔF_MID_, C_10_, and EC_50_ are defined and reported in Table 1 for panels B, C, and D. Data is presented as mean ± SEM, n = 3, *p < 0.05.

**Figure 7:**
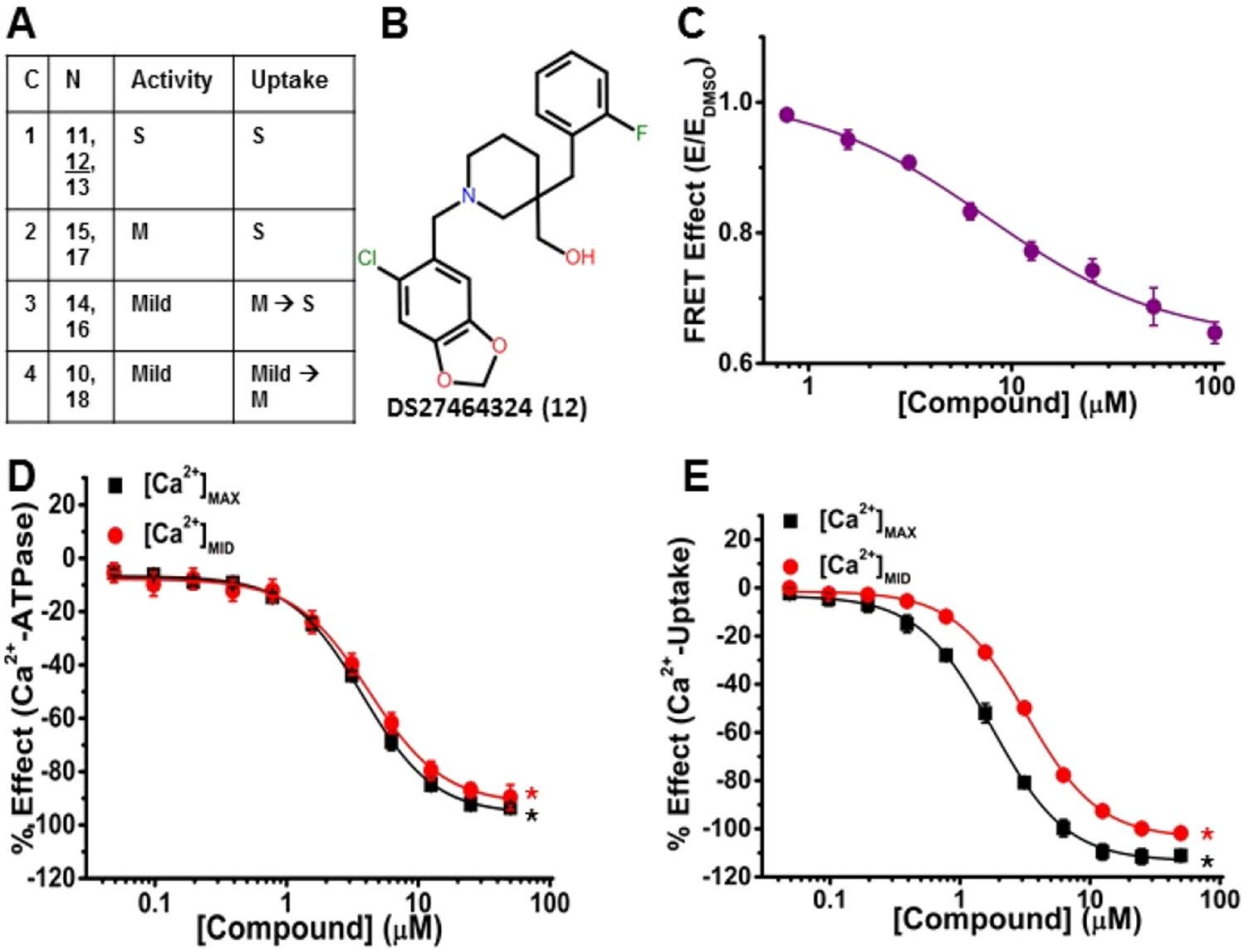
A representative inhibitor (Compound 12) that strongly decreases FRET and inhibits both Ca^2+^-ATPase activities and Ca^2+^-transport. (**A**) Table showing the four subcategories (**C**) of inhibitors (N, Compounds 11–18) and their effects on the Ca^2+^ATPase activity and Ca^2+^-uptake as strong (S), moderate (M), or mild. (**B**) Chemical structure of DS27464324 (Compound 12). (**C**) CRC of normalized FRET E shows decreasing FRET in response to [Compound 12] in 2CS biosensor in live HEK cells. (D) CRC shows inhibition of Ca^2+^-ATPase activity in pCSR under both [Ca^2+^]_MAX_ (black) and [Ca^2+^]_MID_ (red). (**E**) CRC shows inhibition of Ca^2+^-uptake in pCSR under [Ca^2+^]_MAX_ (black) and [Ca^2+^]_MID_ (red). ΔF_MAX_, ΔF_MID_, C_10_, and EC_50_ are defined and reported in Table 1 for panels C, D, and E. Data are presented as mean ± SEM, n = 3, *p < 0.05.

To further evaluate the 18 hit compounds, we determined ΔFLT for [compound] = 0.78–100 μM (Fig. 1B, step 5) in live HEK cells. All 18 hit compounds (Table 1) decreased FRET (increased FLT) of 2CS relative to 1CS (donor-only biosensor), suggesting a structural change (Fig. 1A) in the cytosolic headpiece of SERCA2a. Compounds **1** and **4** showed a significant decrease in FRET at the lower [compound] with no further effect at higher [compound]; testing was not done at even higher [compound] where non-specific effects are likely to dominate. The remaining 16 compounds decreased FRET efficiency (E) with measurable FRET-EC_50_ (Fig. 4 and 7C, Fig. 5 and 6B, and Table 1).

**Table 1:** Results from the concentration response curves (CRC) for 18 hit compounds using FRET, Ca^2+^-ATPase activity, and Ca^2+^ transport assays.

N	C	FRET	Ca^2+^-ATPase						Ca^2+^-Uptake						CR
			[Ca^2+^]_MAX_ (pCa5.4)			[Ca^2+^]_MID_ (pCa6.2)			[Ca^2+^]_MAX_ (pCa5.4)			[Ca^2+^]_MID_ (pCa6.2)			
		EC_50_ (µM)	ΔF_MAX_ (%)*	C_10_ (µM) **	EC_50_ (µM)	ΔF_MID_ (%)*	C_10_ (µM)**	EC_50_ (µM)	ΔF_MAX_ (%)*	C_10_ (µM) **	EC_50_ (µM)	ΔF_MID_ (%)*	C_10_ (µM) **	EC_50_ (µM)	V_U_/V_A_****
1	A	–	**8 ± 6.3**	–	–	**8 ± 5.8**	–	–	*−* *10 ± 2.3*	50	–	**2 ± 1.0**	*–*	–	0.49
2	A	1.4 ± 0.5	**16 ± 3**	48	–	**7 ± 3**	–	–	**4 ± 6.1**	–	–	**4 ± 2.1**	*–*	–	0.53
3	A	1.6 ± 0.2	**21 ± 5.1**	11.0	8.4 ± 3.1	**30 ± 11**	14.8	–	*−* *20 ± 4.2*	17.5	–	**6 ± 3.3**	–	–	0.39
4	B	–	**10 ± 5.3**	50.0	15 ± 7.2	**11 ± 6.1**	39.2	–	**0.5 ± 3.2**	–	–	**11 ± 2.7**	47	–	0.51
5	B	1.5 ± 0.1	**25 ± 3.3**	22.7	–	**35 ± 8.2**	19.6	–	*−* *2 ± 4.2*	–	–	**20 ± 3.3**	22.5	–	0.47
6	E	7.1 ± 0.2	**25 ± 6.3**	9.7	11 ± 1.4	**30 ± 10**	8.7	11.1 ± 1.5	*−* *21 ± 3.3*	15.1	16 ± 1.2	**17 ± 1.4**	4.2	2.9 ± 1	0.48
7	F	0.3 ± 0.1	**14 ± 2**	25	–	**7 ± 3**	–	–	**24 ± 10**	14	–	**19 ± 9.8**	20.6	–	0.74
8	G	4.9 ± 0.3	**49 ± 4.8**	4.3	8.3 ± 0.9	**31 ± 14**	7.8	10.0 ± 1.9	**10 ± 6.5**	25	–	**7 ± 3.0**	*–*	–	0.40
9	C	0.6 ± 0.1	**21 ± 5**	30.7	24 ± 9	**10 ± 6**	–	–	**5 ± 2.2**	–	–	**3 ± 2.4**	*–*	–	0.51
10	C	5.6 ± 0.6	*−* *31 ± 3.2*	7	–	*−* *24 ± 4.0*	3.2	–	*−* *57 ± 3.2*	4.1	–	*−* *34 ± 1.0*	7.9	–	0.35
11	D	14 ± 6.4	*−* *61 ± 1.6*	0.8	3.2 ± 0.4	*−* *59 ± 6.9*	1.2	3.6 ± 0.5	*−* *95 ± 3.7*	0.2	1.8 ± 0.04	*−* *79 ± 1.3*	0.5	2.8 ± 0.1	0.13
12	D	7.6 ± 1.6	*−* *93 ± 1.7*	1.0	3.8 ± 0.3	*−* *90 ± 4.7*	1.0	4.2 ± 0.4	*−* 111 ± 3***	0.4	1.8 ± 0.1	*−* 102 ± 2***	0.8	3.2 ± 0.1	*−* 1.22
13	D	9.9 ± 2.1	*−* *81 ± 2.7*	1	–	*−* *72 ± 3.9*	1.6	–	*−* 112 ± 4***	0.5	3.6 ± 0.1	*−* *97 ± 2*	1.0	6.4 ± 0.2	*−* 0.27
14	H	9.2 ± 1.1	*−* *13 ± 9.7*	6.3	13 ± 11	*−* *26 ± 6.0*	3.2	8.9 ± 2.2	*−* *83 ± 3.5*	1.3	3.4 ± 0.2	*−* *62 ± 1.1*	1.7	3.9 ± 0.2	0.16
15	I	16 ± 4.7	*−* *50 ± 1.3*	0.7	–	*−* *52 ± 5.2*	0.4	–	*−* *85 ± 1.7*	0.9	–	*−* *70 ± 1.4*	1.7	–	0.19
16	J	5.6 ± 0.3	*−* *8 ± 6.3*	0.5	–	*−* *16 ± 8.0*	4.7	–	*−* *66 ± 6.3*	1.2	–	*−* *48 ± 1.8*	4.2	–	0.22
17	K	32 ± 5.3	*−* *52 ± 3.0*	6.2	–	*−* *51 ± 8.2*	5	–	*−* *95 ± 3.1*	1	–	*−* *84 ± 1*	2.2	–	0.09
18	L	3.0 ± 0.3	*−* *24 ± 3.9*	2.2	–	*−* *16 ± 6*	1	0.18 ± 0.2	*−* *41 ± 1.4*	8.6	–	*−* *16 ± 1.5*	22.7	–	0.49

FRET CRC assays were measured using live HEK cells. Ca^2+^-dependent ATPase and Ca^2+^-uptake CRC assays were carried out using pCSR.

N = Numeric compound code used in this paper (manufacturer’s designations are in Supplementary Fig. S2 and Supplementary Table S1). Compounds 1–9 are activators and Compounds 10–18 are inhibitors,

C = Cluster designation of compounds that have a common or unique scaffold that was determined from physicochemical analysis (Supplementary Table S1).

*ΔF_MAX_ and ΔF_MID_ = change in the maximal compound effect (% vs DMSO control) on function (F) (Ca^2+^-uptake rate or Ca^2+^-ATPase rate) observed at a specific Ca^2+^ concentration ([Ca^2+^]_MAX_ (pCa 5.4) or [Ca^2+^]_MID_ (pCa 6.2)) (Fig. 5C).

**C_10_ = Compound concentration yielding 10% effect above or below control (uncertainties similar to those for EC_50_), determined when CRC achieve saturation.

*** = Inhibition value ~ 100%. With strong inhibitors of SERCA2a (e.g., thapsigargin), apparent inhibition > 100% were sometimes observed, probably due to SR leak under control conditions.

EC_50_ = Compound concentration at 50% of the maximum effect. (“–”: insufficient data to define EC_50_ or C_10_.)

****CR = Coupling ratio = V_U_/V_A_, where V_U_ and V_A_ are the maximum values of Ca^2+^-uptake rate and Ca^2+^-ATPase rate, observed at [Ca^2+^]_MAX_. DMSO control = 0.66. SEM ranges from 0.01–0.06.

Bold indicates increase, Italic indicates decrease due to compound.

Mean ± SEM, n = 3, p < 0.05. Examples of representative CRC curves are in Fig. 4–7.

### Functional CRC of hit compounds

To assess the impact of hit compounds on SERCA2a function, we used an absorbance-based Ca^2+^-ATPase activity assay and a fluorescence-based Ca^2+^-uptake assay, using pig cardiac SR (pCSR) vesicles enriched for SERCA2a^23^ (Fig. 1B, step 5). These activities were measured at [Ca^2+^]_MAX_ (saturating, pCa 5.4), [Ca^2+^]_MID_ (subsaturating, midpoint, pCa 6.2), and [Ca^2+^]_BAS_ (basal, pCa 8.0). For the Ca^2+^-ATPase activities, the values at [Ca^2+^]_BAS_ were < 10% of those at [Ca^2+^]_MAX_ and < 25% of those at [Ca^2+^]_MID_, whereas for Ca^2+^-uptake rates, values at [Ca^2+^]_BAS_ were negligible. Functional data acquired at [Ca^2+^]_MAX_ and [Ca^2+^]_MID_ were corrected by subtracting the appropriate basal rate at pCa 8.0 and the % effect due to the compound was fitted to the Hill’s function (Fig. 4–7) to yield values in Table 1. These functional results (Table 1) at [Ca^2+^]_MAX_ and [Ca^2+^]_MID_ were adequate to identify activators (Compounds **1–9**) and inhibitors (Compounds **10–18**) (Table 1 and Fig. S2), based on the functional potency (1/EC_50_, where EC_50_ is the compound concentration at 50% of the maximum effect) and functional efficacy (ΔF_MAX_ or ΔF_MID_; change in the maximal compound effect on function F). Under ideal conditions, a maximum coupling ratio (CR) of 2 Ca^2+^ transported per molecule of ATP hydrolyzed has been reported^30,33–35^. CR defines the efficiency of Ca^2+^ uptake by SERCA, which was determined from the ratio of the measured maximal Ca^2+^-uptake rate (V_U_) at [Ca^2+^]_MAX_, to the maximal Ca^2+^-ATPase activity (V_A_) at [Ca^2+^]_MAX_ (V_U_/V_A_) (Table 1 and under **SERCA2a Activators**). CR was also used to classify and prioritize compounds. Activators were compounds that increased Ca^2+^-ATPase activities and/or Ca^2+^-uptake (at one or both [Ca^2+^]) (Table 1, Fig. 4–6) and were grouped in three categories: (1) increases both Ca^2+^-ATPase activity and Ca^2+^-uptake to increase CR (Compound **7**), (2) increases Ca^2+^-ATPase activity more than Ca^2+^-uptake to decrease CR (Compounds **2**, **4**, **8,** and **9**), and (3) increases Ca^2+^-ATPase activity but inhibits (induces divergent effects on) Ca^2+^-uptake (Compounds **1**, **3**, **5**, and **6**). We define “divergent” to indicate that the compound induces opposing effects at two different [Ca^2+^] (an increase at one [Ca^2+^] and a decrease at the other) in one assay.

Inhibitors induced strong (≥ 68%), moderate (34 to 67%), and mild (≤ 33%) inhibition of SERCA2a and were defined in four subcategories: 1) strong inhibition of Ca^2+^-ATPase activities and Ca^2+^-uptake rates (Compounds **11**, **12**, **13**), 2) moderate inhibition of Ca^2+^-ATPase activities and strong inhibition of Ca^2+^ -uptake (Compounds **15** and **17**), 3) mild inhibition of Ca^2+^-ATPase activities and moderate-to-strong inhibition of Ca^2+^-uptake (Compounds **14** and **16)**, and 4) mild inhibition on Ca^2+^-ATPase activities and mild-to-moderate inhibition on Ca^2+^-uptake (Compounds **10** and **18**) (Table 1, Fig. 7, and under **SERCA2a Inhibitors**).

### Classification of compounds by physicochemical characteristics

The 18 hit compounds were subjected to cheminformatic analysis, to determine whether any shared common chemical scaffolds. Compounds with a Tanimoto coefficient and maximum common substructure (MCS)^36^ scores above 0.4 were binned as clusters, while those with scores below 0.4 were classified as singletons. The analysis yielded diverse scaffolds^36,37^ of hit compounds (Supplementary Fig. S2 and Supplementary Table S1).

Four clusters of compounds (A–D in Table 1) were found, and the remaining eight were unique (singletons) (E–L in Table 1 and Supplementary Fig. S2 and Supplementary Table S1). Compounds in cluster A (**1**, **2**, and **3**) have a common 5-(aryloxymethyl)oxazole-3-carboxamide)^38^; those in cluster B (**4** and **5**) share a N-heteroaryl-N-alkylpiperazine. Cluster C (**9** and **10**) is defined by an amide linkage and cluster D (**11**, **12**, and **13**) by a piperidine scaffold. Clusters E-L (**6, 7**, **8**, **14**, **15**, **16**, **17**, and **18**) contain a singleton with no common scaffold with any other hit compound in this study. All hit compounds have physicochemical properties^39^ that are conducive of favorable drug disposition in vivo, including low molecular weight (< 500), low cLogP (calculated partition coefficient for lipophilicity < 5), low non-H rotatable bonds that describe the molecular flexibility (< 10), low probability of hydrogen bond formation (total number of hydrogen bond acceptors and donors less than 8), and low total polar surface area (tPSA < 140 Å) (Supplementary Table S1).

### SERCA2a activators

Activators (Compounds **1**–**9**) induced varying effects on SERCA2a function (Fig. 4A). In the first category, Compound **7 (**Fig. 4B**)** decreased FRET of 2CS in live cells with FRET-EC_50_ = 0.3 μM, suggesting stabilization of an open conformation of SERCA2a. It accelerated Ca^2+^-ATPase activity at both [Ca^2+^] to induce ΔF_MAX_ = 14% and ΔF_MID_ = 7% (Fig. 4D and Table 1). Compound **7** induced the highest increases in Ca^2+^-uptake of all compounds at both [Ca^2+^] – ΔF_MAX_ (24%) and ΔF_MID_ (19%) (Fig. 4E), which were greater than the increases in the Ca^2+^-ATPase activity (Fig. 4D). CR increased to 0.74 compared to control (0.66, Table 1), indicating increased efficiency of SERCA2a, as needed to increase rate of relaxation and improve contractile function in the heart. Saturation of both CRCs was not achieved at the highest [compound] measured, so the functional EC_50_ was not determined. Instead, we determined C_10_, the [compound] that increases function by 10%. C_10_ was 25 μM for Ca^2+^-ATPase activity at [Ca^2+^]_MAX_ and was not determined at [Ca^2+^]_MID_ because Compound **7** did not induce changes higher than 10%. At [Ca^2+^]_MAX_ and [Ca^2+^]_MID_, C_10_ was 14 μM and 21 μM for Ca^2+^-uptake, respectively (Table 1). This *lead compound* will be prioritized for future optimization by medicinal chemistry, to lower its FRET-EC_50_ and determine functional-EC_50_.

In the second category of activators (Compounds **2**, **4**, **8**, and **9**), Compound **8** (singleton G) (Fig. 5A) decreased FRET (FRET-EC_50_ = 4.9 μM, Table 1, Fig. 5B) and increased Ca^2+^-ATPase activities (ΔF_MAX_ = 49%, the largest increase observed in this study, and ΔF_MID_ = 31%) (Table 1 and Fig. 5C). For Ca^2+^-uptake, the effects were smaller (10% for ΔF_MAX_ and 7% for ΔF_MID_, Fig. 5D), decreasing CR from 0.66 to 0.4 (Table 1). In cardiomyocytes, this should result in a small increase in SR Ca^2+^ uptake, with a greater increase in ATP hydrolysis, resulting in futile cycling of ATP. EC_50_ values for Ca^2+^-ATPase activity were not significantly different at [Ca^2+^]_MAX_ and [Ca^2+^]_MID_ (8.3 and 10 μM, respectively), and were ~ 2 × greater than the FRET-EC_50_ (4.9 μM). C_10_ was 4.3 M ([Ca^2+^]_MAX_) and 7.8 μM ([Ca^2+^]_MID_), indicating significant ATPase activation at low dosage. C_10_ for uptake at [Ca^2+^]_MAX_ was 25 μM and was not determined at [Ca^2+^]_MID_ because Compound **8** did not induce changes higher than 10%.

Compound **2 (**cluster A) induced similar Ca^2+^-ATPase activation (ΔF_MAX_ = 16%, ΔF_MID_ = 7%) as Compound **7**, with slightly smaller increases in Ca^2+^-uptake (Table 1), decreasing CR from 0.66 to 0.53. Compound **4** (cluster B) increased Ca^2+^-ATPase activities at both [Ca^2+^]_MAX_ and [Ca^2+^]_MID_ by ~ 10%, and increased Ca^2+^-uptake at both [Ca^2+^]_MAX_ (0.5%) and [Ca^2+^]_MID_ (11%). Compound **9** (cluster C) increased ΔF_MAX_ (21%) and ΔF_MID_ (10%) for Ca^2+^-ATPase activity, though with smaller increases for Ca^2+^-uptake (3–5%). Thus, Compounds **4** and **9** decreased CR similarly (Table 1), indicating that SERCA2a transport efficiency was diminished slightly. Compounds **2**, **4**, **8**, and** 9** increased Ca^2+^ uptake (0.5–10%). Thus these four compounds join Compound **7** as *promising future lead compounds* (Fig. 1B).

In the third category, Compounds **1** and **3** (cluster A), **5** (cluster B), and **6** (singleton E) increased Ca^2+^-ATPase activities at both [Ca^2+^]_MAX_ and [Ca^2+^]_MID_, but induced divergent (inhibitory) effects on Ca^2+^-uptake at [Ca^2+^]_MAX,_ decreasing CR (0.39—0.48). In cardiomyocytes, this decrease in CR would likely result in a decrease in SR Ca^2+^-uptake, while increasing ATP hydrolysis. Compound** 6** (Fig. 6A) decreased FRET with FRET-EC_50_ = 7.1 μM (Fig. 6B, Table 1), while moderately increasing Ca^2+^-ATPase activity (ΔF_MAX_ = 25% and ΔF_MID_ = 30%) (Fig. 6C), with functional EC_50_ = 11 μM at both [Ca^2+^]. It induced divergent effects on Ca^2+^-uptake, decreasing ΔF_MAX_ by 21% and increasing ΔF_MID_ by 17% (Table 1 and Fig. 6D), but had inhibitory effects at high [compound], typical of hormesis^40^ (biphasic dose response), which can disrupt Ca^2+^ homeostasis^41^. C_10_ was similar at both [Ca^2+^] (9.7 μM and 8.7 μM), but was significantly different for Ca^2+^-uptake (15 μM at [Ca^2+^]_MAX_, 4 μM at [Ca^2+^]_MID_), decreasing CR to 0.48.

Compound **5** increased Ca^2+^-ATPase activity moderately at [Ca^2+^]_MAX_ (25%) and [Ca^2+^]_MID_ (35%). Ca^2+^-uptake was inhibited slightly at [Ca^2+^]_MAX_ (2%), but activated at [Ca^2+^]_MID_ (20%) (Table 1). Compounds **1**, and **3** induced low activating effects at [Ca^2+^]_MID_ for Ca^2+^-uptake (2% and 6%), but they inhibited Ca^2+^-uptake at [Ca^2+^]_MAX_ (10% and 20%) (Table 1). Compounds **1** and **5** induced similar decreases in the CR (to 0.49 and 0.47), while Compound **3** induced a slightly smaller CR of 0.39 (Table 1). These effects are similar to those of unphosphorylated PLB in cardiac SR^42^.

Compounds **2**, **7**, **8**, and **9** induced Ca^2+^-dependent activation of the Ca^2+^-ATPase activity over the range of [compound] studied. Compounds **1**, **4**, and **6** showed similar activation of Ca^2+^-ATPase activity at the two [Ca^2+^] measured, while Compounds **3** and **5** showed higher activation at [Ca^2+^]_MID_ compared to [Ca^2+^]_MAX_.

### SERCA2a Inhibitors

Although our primary goal is to find SERCA2a activators for treatment of HF, it has been proposed that SERCA2a inhibitors or uncouplers could be effective for treating several diseases, such as cancer and malaria^17,18^. Compounds **10**–**18** decreased Ca^2+^-ATPase activities and Ca^2+^-transport at [Ca^2+^]_MAX_ and [Ca^2+^]_MID_ to varying extents; strong (S), moderate (M), or mild and are in four subcategories (Fig. 7A). Compared with FRET-EC_50_ of the activators (0.3–7 μM), most of the inhibitors (Compounds **10**–**18**) showed weaker affinity, with FRET-EC_50_ values in the range of 5–32 μM, but the maximum inhibitory effects (efficacies) of the inhibitors tended to be greater (Table 1).

(1) Compounds **11**, **12**, and **13** (cluster D) showed similar inhibition of both SERCA2a functions, except that, compared with the Ca^2+^-ATPase activity, Ca^2+^-uptake inhibition at [Ca^2+^]_MID_ required slightly higher [compound], as shown by the right-shift of the red curve (Fig. 7D). Compound **12** (cluster D, Fig. 7B) strongly inhibited both Ca^2+^-ATPase activity and Ca^2+^-uptake (Fig. 7D, E) to levels similar to the classic SERCA inhibitor thapsigargin (Tg), although Tg acts with much greater affinity (EC_50_ ≈ 7.5 nM^23^) than Compound **12** (EC_50_ = 3.8 μM, Fig. 7C, Table 1). (2) Compounds **15** (singleton I) and **17** (singleton K) induced moderate inhibition of both activities, decreasing ΔF_MAX_ and ΔF_MID_ by ~ 50% for Ca^2+^-ATPase activity and slightly more for Ca^2+^-uptake (70–95%) (Table 1). (3) Compounds **14** (singleton H) and **16** (singleton J) induced mild inhibition of Ca^2+^-ATPase activity, but a moderate-to-strong inhibition of the Ca^2+^-uptake (Fig. 7A, Table 1). (4) Compounds **10** (cluster C) and **18** (singleton L) induced mild inhibition of Ca^2+^-ATPase and mild-to-moderate inhibition of Ca^2+^-uptake (Fig. 7A, Table 1). All inhibitors decreased CR, the efficiency of Ca^2+^-transport.

## Discussion

We have identified new compounds based on an increase in ΔFLT within a human cardiac 2CS biosensor expressed in live mammalian cells at low [Ca^2+^] (the normal condition in the cytoplasm of HEK cells). This decrease in FRET implies that the actuator (A) and nucleotide-binding domains (N) of SERCA2a moved apart, supporting an open configuration at low [Ca^2+^] in HEK cells, possibly priming SERCA in a more open state to bind Ca^2+^. Previous studies with a different SERCA2a biosensor indicated that the addition of Ca^2+^ induces a decrease in FRET^21^. Our functional assays at high and mid [Ca^2+^] show activating, uncoupling, and inhibiting effects that may correlate with structural changes. Further future elucidation of the compounds’ effects on SERCA2a conformational states will require detailed analysis of FLT-detected FRET and transient kinetics data. We identified three categories of activators that (1) increase both Ca^2+^-ATPase activity and Ca^2+^-transport to increase CR (Compound **7**, Fig. 4) (2) increase Ca^2+^-ATPase activity and Ca^2+^-transport to decrease CR (Compounds **2**, **4**, **8,** and **9,** Fig. 5), and (3) increase Ca^2+^-ATPase activity but inhibits Ca^2+^-transport to decrease CR (Compounds **1**, **3**, **5**, and **6**) (Fig. 6). We identified four subcategories of inhibitors based on the extent of decrease in Ca^2+^ATPase activity and Ca^2+^-transport for SERCA2a (Table 1, Fig. 7A).

Most FRET-EC_50_ values were smaller (higher potency) for activators (Compounds **1–9**; 0.3-7 μM) than for inhibitors (Compounds **10–18**; (3-32 μM) (Table 1). However, the functional C_10_ and EC_50_ values were smaller, indicating greater potency, for inhibitors than for activators. Potencies observed by FRET and function are not precisely correlated, probably because the assays were performed on different types of samples (live cells vs. purified proteins), low nM [Ca^2+^] in live HEK cells^43^ vs. μM [Ca^2+^] in the pCSR in vitro assays), which measure different properties (structure vs function). Functional CRC assays showed that inhibitors tend to induce larger changes (indicating higher efficacy) than activators, in both Ca^2+^-ATPase activity and Ca^2+^-uptake. Most inhibitors induced a larger change in Ca^2+^-uptake than in Ca^2+^-ATPase activity, decreasing CR.

Most of the activators reduced CR, inducing larger changes in Ca^2+^-ATPase activity than in Ca^2+^-uptake. A notable exception is Compound **7**, which increases Ca^2+^-transport even more than it increases Ca^2+^-ATPase activity, increasing CR. This compound will be a *high priority as a lead compound* for future efforts in medicinal chemistry and assays of physiological function. Compounds **2**, **4**, **8**, and **9** will have only slightly lower priority.

Ten compounds were binned into four clusters (A–D); eight were singletons (E–L) (Table 1). Many compounds showed similar functional traits, suggesting that ligand-sensing sites in SERCA2a are recognized by a range of scaffolds, or that these sites are close to each other, providing potentially powerful tools in the design of future compounds^44–46^. Only Compound **7** (singleton F) induced higher activation in Ca^2+^ transport than in the Ca^2+^-ATPase activity. Compounds **2** (cluster A), **4** (cluster B), **8** (singleton G), and **9** (cluster C) induced similar effects of moderate activation of Ca^2+^-ATPase activity, with smaller activation of Ca^2+^-transport. Compounds in activator clusters A (Compounds **1** and **3**) and B (Compound **5**) along with Compound **6** from singleton E, showed similar functional effects: moderate ATPase activation with mild inhibition of Ca^2+^-transport at [Ca^2+^]_MAX_. Inhibitor compounds in clusters D (**11**, **12**, and **13**), C (**10**), and H–L (**14**–**18**) induced a range of effects at both [Ca^2+^], which will be useful in designing derivatives for structure activity relationship (SAR) analysis.

There was negligible overlap in hit compounds identified in our previous FRET-HTS of the DIVERSet-CL targeting tumor necrosis factor receptor 1^27^. There was 81% overlap in the fluorescent compounds detected (and thus rejected) in these two HTS studies, indicating that our FRET-HTS methodologies are effective and versatile^26,29^. In our previous study of the DIVERSet-CL, using the SERCA2a Ca^2+^-ATPase activity as the primary HTS assay (ATPase-HTS), we discovered 19 activators^30^. While no identical activators were found in that ATPase-HTS study^30^ and in the current FRET-HTS study, there were several compounds with similar scaffolds that showed similar functional results. These compounds share the oxadiazol scaffold and activated the Ca^2+^-ATPase activity but inhibited Ca^2+^-uptake. Another common scaffold is the amide group; six compounds^30^ identified with an amide induced a smaller increase in the Ca^2+^-transport than in the Ca^2+^-ATPase activity, reducing CR similar to Compound **9** in this study. It is not surprising that the two studies did not identify the same compounds, because (a) the FRET-HTS assay was performed with human cardiac 2CS in live HEK cells in low [Ca^2+^], while the ATPase-HTS assay was done in purified SR from rabbit skeletal muscle (SERCA1a) under high [Ca^2+^]^30^, (b) the FRET assay is much more precise than the functional assay, (c) the relationship between SERCA structure and function is complex, and (d) the binding sites on SERCA2a for these compounds are unknown. As discussed above, a ligand-binding site may recognize several different scaffolds^44–46^. It is also possible that a compound binds to PLB or competes with PLB for binding to SERCA, thus increasing SERCA2a activity, as was shown for the activator, istaroxime^47,48^. These observations highlight the value of complementary HTS assays for the same target.

Activation of Ca^2+^-transport by SERCA2a is needed when cardiac relaxation is impaired, as in diastolic dysfunction^1^ or diabetic cardiomyopathy^49^. SERCA2a activation is a promising strategy, in combination with current drugs such as β-blockers and ACE inhibitors^50^. Activation of SERCA also has therapeutic potential for Alzheimer’s disease^51^ or Duchenne muscular dystrophy (DMD)^52^. Until recently, very few compounds were known to stimulate SERCA2a: CDN1163 (stimulates Ca^2+^ transport)^24,53^, CP-154526 (increases the apparent Ca^2+^ affinity of SERC2a)^54^, Ro 41–0960 (increases SERCA2a maximal activity in high Ca^2+^)^54^, and istaroxime (stimulates SERCA2a activity)^55^. However, our recent ATPase-HTS assay identified ~ 19 new activators of SERCA^30^, and we identified nine in the present study. A SERCA activator from our previous work (CDN1163) shows promise as a therapeutic agent for Alzheimer’s disease^51^ and for DMD^52^. Of all these SERCA2a activators, only istaroxime has been in phase IIb clinical trials for treatment of heart failure^55,56^. However, because of its unsuitability for human usage^56^, istaroxime must be modified^47,48^.

Compounds **1, 3, 5**, and **6** induced small effects on the Ca^2+^-ATPase activity (~ 10–25% increase) and induced a negative effect on the Ca^2+^-transport (Fig. 6C and D), thus decreasing the CR, which is likely to increase heat output^15,16,57^. These effects are similar to that of SLN on SERCA1a (skeletal muscle), where SLN reduces Ca^2+^-transport without affecting the Ca^2+^-ATPase activity (SERCA1a uncoupling), thus reducing CR^15^. Uncoupling of SERCA1a leads to higher usage of ATP, which enhances non-shivering thermogenesis (NST)^15^. Another contributor to NST is Ca^2+^ leak from SR through resting RyR channels, stimulating SERCA to re-sequester Ca^2+^ into SR, thus using more ATP and generating heat^58^, which has been suggested as a potential therapeutic strategy for reducing obesity^15,57^.

Six decades of research for SERCA inhibitors as oncology therapeutics have yielded hundreds of SERCA inhibitors with varying potencies and efficacies^17^. Similarly, our discovery of new SERCA inhibitors with a range of potencies and efficacies is likely to be advantageous for non-cardiac applications^17,18^.

Here we successfully used the 2CS biosensor to identify novel small-molecule effectors of SERCA with diverse chemical scaffolds, resulting in an array of activator and inhibitor hit compounds. Most importantly, based on the amplitude of the functional effects on SERCA2a, we discovered *a potential lead compound* (Compound **7**) that activates Ca^2+^-uptake more than the Ca^2+^-ATPase activity, increasing the CR, so this will be a *high priority* for future efforts in medicinal chemistry and assays of physiological function, along with four other promising SERCA2a activators. The innovative technology included two novel plate-readers – the FLT instrument used in the primary screen, and a spectral instrument – that were used to remove compounds with interfering fluorescence signals, allowing us to focus on valid SERCA activators and inhibitors. It is possible that some of the eliminated fluorescent compounds have potential as SERCA2a effectors, which could be evaluated in future work using our ATPase activity assay^30^. In future studies, we will evaluate these hit compounds in more functional detail, including the full range of [Ca^2+^], SERCA isoforms, Na^+^/Ca^2+^ exchanger, RyR, and L-type Ca^2+^-channels. Medicinal chemistry will be done to elucidate SAR and to design analogs with greater potency and specificity^27,59^, justifying studies in intact muscles and animals. Finally, we have shown that our primary screening technology can perform precise HTS on several thousand compounds per hour, making this approach capable of application on an industrial scale, screening millions of compounds.

## Methods

### Molecular biology

A two-color intramolecular human SERCA2a (2CS) biosensor, based on human cardiac SERCA2a fused to green fluorescent protein (eGFP) and red fluorescent protein (tagRFP) was developed to detect structural changes that are related to the functional changes of SERCA^20,22,25^. Briefly, tagRFP was genetically fused to the N-terminus of SERCA (A-domain) and eGFP was inserted as an intrasequence tag before residue 509 in the nucleotide-binding domain (N-domain)^60,61^. Donor-only and acceptor-only (1CS) biosensors were created in a similar manner as the 2CS biosensor but with the construct containing either only eGFP or only tag-RFP, respectively. The fluorescent proteins fused to SERCA in 2CS and 1CS do not significantly affect SERCA activity, in membranes purified from HEK cells^23,25^. A null-biosensor construct consisting of eGFP and tagRFP connected by a 32-residue unstructured flexible linker peptide (G32R) was created as described previously^23,25^. All constructs were cloned into expression vectors containing the genes for antibiotic resistance to G418, puromycin, or blasticidin.

### Cell culture

Stable cell lines were generated using either human embryonic kidney (HEK) HEK293 (ATCC, Manassas, VA) or HEK293-6E (National Research Council, Canada) cells^25^. Briefly, cells were transiently transfected with 2CS, 1CS, or G32R null-biosensor plasmids using Lipofecatime 3000 or 293fectin (Thermo Fisher Scientific). Flow cytometry was used to select and enrich for the population of cells expressing respective biosensors. Stable HEK293 cell lines were maintained in phenol red-free DMEM media (Gibco, Waltham, MA) supplemented with 2 mM GlutaMAX (Gibco, Waltham, MA), 10% fetal bovine serum (FBS) (Atlanta Biologicals, Lawrenceville, GA), 1 IU/mL penicillin/streptomycin (Gibco, Waltham, MA), and 250 μg/mL G418 (Fisher Scientific). Stable HEK293-6E cell lines were maintained in F17 media (Sigma Aldrich) supplemented with Kolliphor p188 (Sigma Aldrich, St. Louis, MO), 200 nM/mL GlutaMAX, and either 1 μg/mL puromycin (Invitrogen, Carlsbad, CA) or 2 μg/mL blasticidin (Goldbio) as a selection antibiotic. All cell lines were grown at 37 °C with 5% CO_2_.

### Compound handling

A 50,000 DIVERSet-CL was purchased from ChemBridge Corporation (San Diego, CA) at a 10 mM stock concentration for each compound. All compounds met the high quality standard of 100% identification by NMR and/or LC–MS and have a minimum purity of 85% and their identity verified using LC–MS/ELSD as confirmed by the ChemBridge Corporation. For the FLT HTS initial screens, the compound library was reformatted into 384 well Echo compatible plates using the Biomek FX (Beckman Coulter, Miami, FL) and 5 nL of either compound (columns 3–22 and 27–46) or DMSO (columns 1–2, 23–26, and 47–48) was dispensed into forty 1536 well black polystyrene assay plates (Greiner, Kremsmünste, Austria) using an Echo 550 liquid dispenser (Beckman Coulter) to yield a final assay screening concentration of 10 μM. The low autofluorescence and low interwell cross-talk of these plates made them advantageous for FLT measurements. Plates were heat sealed with a PlateLoc Thermal Microplate Sealer (Agilent, Santa Clara, CA) and stored at − 20 °C prior to use. The same methods were applied for subsequent FLT retesting of the hit compounds identified in the FLT screen (Fig. 1B, steps 1–3), except that the [compound] was tested at 10 μM and 30 μM in triplicate, where the latter gave more reproducible results.

FRET CRC assay plates (0.78–100 μM compound range) with at least ten different compound concentrations were made by adding the appropriate volume of compound or DMSO into black 384 well plates (Greiner Bio-One) using a Mosquito HV (SPTLabTech, United Kingdom). Subsequent Ca^2+^-ATPase activity and Ca^2+^-transport CRC assay plates (0–50 μM compound range) with repurchased compounds were made in a similar manner using with the Echo 550 (Beckman Coulter) using either 384 well transparent plates (Greiner Bio-One) or black-walled plates with transparent bottoms (Greiner Bio-One), respectively.

### HTS sample preparation and FRET measurements

On each day of screening, cells were harvested, washed three times with PBS, and centrifuged at 300* g* for 5 min. Cells were filtered using a 70 µm cell strainer and diluted to 1–2 × 10^6^ cells/mL. Cell concentration and viability were assessed using the Cell countess (Invitrogen) and trypan blue assay. During assays, cells were constantly and gently stirred using a magnetic stir bar at room temperature, keeping the cells in suspension and evenly distributed to avoid clumping. HEK 293-cells expressing 2CS were dispensed at 5 μL or 50 μL per well into assay plates (dispensed into 40 assay plates, each containing 1536 wells) pre-plated with compounds (from a DIVERset 50,000 compound library) using a Multdrop Combi liquid dispenser (Thermo Fisher Scientific, Pittsburg, PA) and sealed until needed. Because the kinetics of membrane permeability, diffusion, and/or binding of the compound to live cells may be compound-dependent, we tested two incubation times, 20 min and 120 min, for the FRET CRC. FRET EC_50_ values determined from both incubations were similar, but the 120 min incubation yielded a more reproducible and sigmoidal curve. Plates containing eight-point concentration curves of three tool compounds (known SERCA effectors thapsigargin, BHQ, and CPA)^22^ were used as positive controls for biosensor function and performance prior to running the full-scale FRET-HTS assay.

The primary FLT-FRET-HTS assay was performed over two days with a custom HTS fluorescence lifetime plate reader (FLT-PR) with donor emission detected at 517 nm (Fig. 1B, step 1). Initial hits from this FLT screen were selected (using rZ-score, discussed below), then fluorescent compounds were removed using the Similarity Index (SI) calculated from the spectral measurement acquired with the SUPR (SI, discussed below) (Fig. 1B, step 2). Both instruments were provided by Photonic Pharma LLC (Minneapolis, MN)^23^.

The same methods were applied for subsequent FRET retesting (Fig. 1B, step 4) of the reproducible hit compounds identified in Fig. 1B, steps 1–3, except that the compounds were tested at 10 μM and 30 μM [compound]. 160 hit compounds were selected from the library master plates and reloaded onto new assay plates for retesting with 2CS and a null-biosensor, using FLT-PR (ΔFLT) and SUPR (Δ(G/R)). This step was designed to remove compounds that bind directly to the fluorescent protein or produce other artifacts in the FLT reading that do not involve FRET. Then 18 hit compounds, representing a range of ΔFLT, were selected and purchased from ChemBridge to determine CRC from FRET, Ca^2+^-ATPase activity, and Ca^2+^-transport assays using at least ten different concentrations by repeatedly scanning the 1536-well plates.

### FRET-HTS instrumentation and data analysis

A detailed description of the high-throughput fluorescence lifetime plate reader (FLT-PR) and spectral unmixing plate reader (SUPR), manufactured by Fluorescence Innovations Inc and provided by Photonic Pharma, LLC was described previously^23,26^. Briefly, for lifetime measurement with the FLT-PR, the observed donor-fluorescence waveform, I(t) was fit by a convolution of the measured instrument response function (IRF) (Eq. 1a) and a single-exponential decay F(t) to obtain the lifetime (τ) of the donor fluorophore^22,26,62^ in the absence (τ_D_) and presence (τ_DA_) of the acceptor as described in Eq. (1b):

1a $$I\left(t\right)=F\left(t\right)*IRF$$

1b $$F\left(t\right)=A {e}^{(- \frac{t}{\tau })}$$

In experiments with a donor-only control, FRET efficiency (*E*) was determined as the fractional decrease of donor FLT in the absence and in the presence of acceptor as in Eq. (2):

2 $$E=1- \frac{{\tau }_{DA}}{{\tau }_{D}}$$

*E* was determined in the presence and absence of compound and normalized relative to *E* of the DMSO control. For spectral detection of FRET with SUPR, the observed fluorescence emission spectrum F(λ) was fit by least-squares minimization of a linear combination of component spectra for donor (G for green), acceptor (R for red), cellular autofluorescence (C) and water Raman (W), as described previously^22^. The change in ratio of the mole fractions of the G and R component spectra between compound and DMSO control (Δ(G/R)) provides a direct indication of a change in FRET due to biosensor structural changes, independent of the lifetime measurements. Together, these complementary metrics provide an effective method for eliminating false positives arising in either method.

### HTS data analysis

FLT-PR data was used as the primary metric for flagging potential hit compounds. After fitting waveforms with a single exponential decay to quantify donor lifetime, the change in fluorescence lifetime due to compound (Δτ) was computed by performing a moving median subtraction in the order the plate was scanned, with a window size of 24 wells, rather than subtracting DMSO controls. The reasons for this are twofold: 1) plate gradients are often observed due to heating of the digitizer during acquisition and 2) performing Δτ computations with DMSO controls can sometimes result in artifacts as a half of the DMSO wells are on the edge of plates, which occasionally exhibit artifacts due to processes needed for the preparation of the compound library being tested. As most compounds are likely to be non-hits, and therefore DMSO like, computation of a moving median is an effective alternative to solving both gradient issues and edge-effect distortion of the primary metric for hit selection, Δτ.

Previous validation in 1536-well plates indicated that the Z' parameter – a measure of HTS assay quality that factors in the signal window and the variance of positive and negative controls (i.e., thapsigargin and DMSO vehicle)^63^ – yielded a value of 0.62^25^. A value of 0.5 ≤ Z' < 1 indicates excellent assay quality, ready for large-scale HTS^63^ as we recently showed for an ATP-based HTS assay^30^. Data from the control (tool) compounds was not needed for assessment of assay quality in the full-scale HTS in this study, instead we used the coefficient of variance (CV), computed using wells containing only DMSO, to assess the quality of the plates, in order to separate within-plate and between-day variability, as in our previous ATP-based HTS study^30^.

In our previous ATPase-based HTS study, we defined a hit compound as one that changes the ATPase signal by 4SD relative to the DMSO controls^30^. Given the increased precision (< 1% CV, Fig. 2A) afforded by the FLT measurement and the availability of complementary HTS measurements using the spectral plate reader on the same plate for further triage, we set our hit threshold to an r*Z*-score of ± 3, on a plate-by-plate basis, in order to include a broader range of initial hit compounds. The rZ-score was used (instead of the standard *Z-*score), where the median (*M*) and median absolute deviation (*MAD*) are used in place of the mean and standard deviation (Eq. 3), to best capture the most hits, as the standard *Z*-score is more susceptible to strong outliers^23^.

3 $${\text{r}}Z{-}{\text{score}}=\frac{\Delta \tau - M(\Delta \tau )}{MAD(\Delta \tau )}$$

To remove interfering fluorescent compounds, the similarity index (SI)^22^ was computed by comparing a region (500–540 nm) of the donor-only spectrum (*I*^(a)^) for each well to that of the plate-wide average DMSO spectrum (*I*^(b)^) in the same wavelength band as described in Eq. 4^26^. Compounds that reproducibly exceeded an SI r*Z*-score of 5 (corresponding to an SI of 2 × 10^–4^) were deemed likely fluorescent compounds and were removed from consideration.

4 $$SI=1- \frac{\sum {I}_{i}^{(a)}. {I}_{i}^{(b)}}{\sqrt{\sum {{I}_{i}^{(a)} . I}_{i}^{(a)}} \sqrt{\sum {{I}_{i}^{(b)} . I}_{i}^{\left(b\right) }}}$$

Spectral (SUPR) data was processed similarly to FLT-PR data, with the Δ(G/R) metric being computed by applying the same moving median filter on the initial measurement of the ratio of donor (G) to acceptor (R) mole fractions (G/R)^21,25^. The hit threshold was also set using an r*Z*-score of ± 3. While the FLT-PR data and SUPR data showed strong correlation, the FLT-PR data exhibited some clear outliers, presumably due to compounds directly modifying the donor lifetime. To eliminate these likely interfering compounds, correlation was enforced by eliminating compounds that exceed an r*Z*-score of ± 3 from the median value of the ratio of Δτ over the Δ(G/R) metric.

### Cardiac SR preparation

Cardiac SR vesicles were isolated from fresh porcine left ventricular tissue using differential centrifugation of the homogenized tissue as previously described^20^. The SR vesicles were flash-frozen and stored at − 80 °C until needed. The SERCA concentration in the ER preparations purified from HEK cell homogenate is at least 10 times less than in purified porcine cardiac SR^20^, but there was sufficient expression of the fluorescent SERCA2a biosensor in HEK cells to detect FRET with high precision by FLT^22,23,26^.

### Effects of hit compounds on the Ca^2+^-ATPase activity of SERCA

Functional assays were performed using porcine cardiac SR (pCSR) vesicles^20^. An enzyme-coupled, NADH-linked ATPase assay was used to measure SERCA ATPase activity in 384-well microplates. Each well contained 50 mM MOPS (pH 7.0), 100 mM KCl, 1 mM EGTA, 0.2 mM NADH, 1 mM phosphoenol pyruvate, 10 IU/mL of pyruvate kinase, 10 IU/mL of lactate dehydrogenase, 7 µM of the calcium ionophore A23187 (Sigma), and CaCl_2_ was added to set free [Ca^2+^] to three different concentrations^23^. Ca^2+^-ATPase activity was measured at three [Ca^2+^]: [Ca^2+^]_MAX_ (saturating, pCa 5.4), [Ca^2+^]_MID_ (subsaturating, midpoint, pCa 6.2), and [Ca^2+^]_BAS_ (basal non-activating, pCa 8.0). 10 μg/mL of SR vesicle, calcium, compound (0.048 to 50 μM), and assay mix were incubated for 20 min at room temperature before measurement of functional assays with each of the 18 hit compounds, because a shorter incubation time than the FRET live-cell assays achieved optimal responses. The assay was started upon the addition of MgATP, at a final concentration of 5 mM (total volume to 80 μL), and absorbance was read at 340 nm in a SpectraMax Plus^384^ microplate spectrophotometer (Molecular Devices, Sunnyvale, CA).

### Effects of hit compounds on the Ca^2+^-transport activity of SERCA

Ca^2+^-transport assays were performed with similar porcine SR samples as in the Ca^2+^-ATPase assays described above. The compound effect on the Ca^2+^-transport activity of SERCA2a was determined using an oxalate-supported assay in which the change in fluorescence in a Ca^2+^-sensitive dye, Fluor-4, was determined as previously described^23^. A buffered solution containing 50 mM MOPS (pH 7.0), 100 mM KCl, 30 mg/mL sucrose, 1 mM EGTA, 10 mM potassium oxalate, 2 M Fluo-4, 30 µg/mL porcine cardiac SR vesicles, CaCl_2_ calculated to reach the free [Ca^2+^] (pCa 8.0, 6.2, and 5.4), and compound (0.048 to 50 μM) was dispensed into 384-well black walled, transparent bottomed plates (Greiner Bio-One) containing the tested small molecule and incubated at 22℃ for 20 min while covered and protected from light. To start the reaction, MgATP was added to a final concentration of 5 mM, and the decrease in 485-nm excited fluorescence of Fluo-4 was monitored at 520 nm for 15 min using a FLIPR Tetra (Molecular Devices, San Jose, CA).

### Data analysis of FRET CRC assays of hit compounds

FRET efficiency (*E*) (Eq. 2) was determined as the fractional decrease of donor lifetime (τ_D_ = 2.5 ± 0.01 ns for 1CS, donor only) in the presence of acceptor (2CS, τ_DA_ = 2.33 ± 0.001 ns) due to FRET. *E* was plotted as “FRET Effect (E/E_DMSO_)” vs [compound], fitted to the Hill’s function for determination of FRET-EC_50_^27,31^. This normalization of E corrects for variation of controls done on different days.

### Data analysis of Ca^2+^-ATPase and Ca^2+^-transport activities from CRC assays

SERCA2a activity, F (rate of ATP hydrolysis or Ca^2+^ uptake), was measured at varying pCa and varying compound concentration. F measured at [Ca^2+^]_MAX_ (saturating, pCa 5.4) or [Ca^2+^]_MID_ (subsaturating, midpoint, pCa 6.2) was corrected by subtracting the basal rate at pCa 8.0) and the % effect due to compound was reported. Concentration response curves (CRC) were fitted using the Hill function to determine V_MAX_ (the activity at saturating [compound]), and EC_50_, the compound concentration at 50% effect^54^. When the CRC did not achieve saturation, the maximal change (Δ) in activity was determined, to yield ΔF_MAX_ and ΔF_MID_ at the [Ca^2+^]_MAX_ and [Ca^2+^]_MID_ conditions, respectively (Fig. 5C, Table 1). ΔF_MAX,_ ΔF_MID_, C_10_ (compound concentration inducing 10% effect), and EC_50_ are reported in Table 1.

### Cheminformatic analysis of hit compounds

An online interactive program was used to perform cheminformatics analysis^64^ to determine whether the hit compounds had structural similarity by identifying common chemical scaffolds (core structural feature) using binning, multidimensional scaling (MDS), and compound similarity methods where the Tanimoto coefficient^36^ and maximum common substructure^36^ values were used to determine clustering (Supplementary Table S1). The physicochemical properties (for e.g. Lipinski Rule of 5) and bioactivity properties of the compounds were also used in the clustering analysis^39^. A cluster contained two or more compounds with similarity score > 0.4, while a unique compound with a similarity score < 0.4 was referred to as a singleton.

### Statistical analysis

Analysis of two-group comparisons was done by a two-tailed unpaired Student’s t-test (*p < 0.05) using the data analysis program Microsoft Excel (Santa Rosa, CA). Data are presented as mean ± SEM calculated from a minimum of three separate experiments (n = 3).

## Supplementary Information

Supplementary Information.

## Data Availability

All the data discussed are presented within the article and Supplementary Information and are available from the corresponding authors (OR and DDT) on reasonable request.
