# Supplementary material for: Fluorescence lifetime FRET assay for live-cell high-throughput screening of the cardiac SERCA pump yields multiple classes of small-molecule allosteric modulators
Source: Sci Rep. 2023 Jul 1;13:10673. doi: 10.1038/s41598-023-37704-x (PMC10314922; doi:10.1038/s41598-023-37704-x)
Supplement: Supplementary file 1 — Supplementary Information. [file 41598_2023_37704_MOESM1_ESM.pdf]

## Supplementary Information

# Fluorescence lifetime FRET assay for live-cell high-throughput screening of the cardiac SERCA pump yields multiple classes of small-molecule allosteric modulators

Osha Roopnarine[1, 4]*, Samantha L. Yuen[1, 4] Andrew R. Thompson[1], Lauren N. Roelike[1], Robyn T. Rebbeck[1], Philip A. Bidwell[2], Courtney C. Aldrich[3], Razvan L. Cornea[1], and David D. Thomas[1]*

[1]*Department of Biochemistry, Molecular Biology and Biophysics, University of Minnesota, Minneapolis, MN, 55455*
[2]*Department of Medicine, Cardiovascular Division, University of Minnesota, Minneapolis, MN 55455*
[3]*Department of Medicinal Chemistry, University of Minnesota, Minneapolis, MN 55455*
[4]*These authors contributed equally*

*Corresponding authors: Dr. Osha Roopnarine and Dr. David D. Thomas
Email: roopn001@umn.edu, ddt@umn.edu

This document contains Supplementary Information for the Results section.

Page S2: Supplementary Fig. S1 showing FLT retests of selected hit compounds with 2CS and null biosensor to support data in Figure 3C and D.

Page S3: Supplementary Fig. S2 showing results from FRET HTS of DIVERSet library in Figure 2 and 3 showing 18 hit compounds.

Page S4: Supplementary Table 1 showing chemical names and physio-chemical properties of 18 hit compounds from Figure S1, resulting from analysis in Figures 2 and 3 in Results.

Page S7: Supplementary References

**Removal of null-biosensor effectors.** The ΔFLT response of compounds with the null biosensor compared to 2CS show distinct correlations, indicating that the observed ΔFLT effects in 2CS cannot be explained by a simple interaction with the donor fluorophore. Compounds that were considered for functional testing exceeded a ≥ 70ps 2CS response, and any compounds that exceeded 50ps response in the null-biosensor were excluded. Coupled with the ΔG/R changes in 2CS that are not observed for the null-biosensor (as shown in Fig. 3C and D), we can be confident that the observed ΔFLT were due to changes in 2CS FRET.

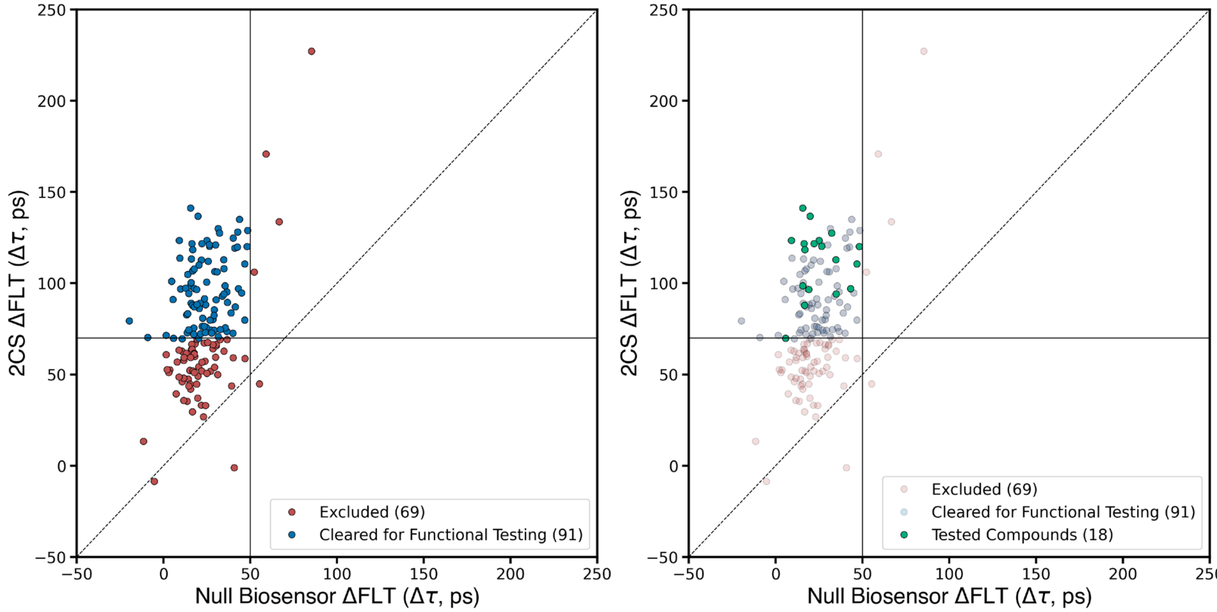


**Supplementary Figure S1. (A)** Plot of ΔFLT compound response of 2CS *versus* the null biosensor showing poor correlation (where a 1:1 correlation would be described by the dashed diagonal line), and indicating that the ΔFLT effects observed with 2CS were not due merely to modification of donor fluorescence by compound interaction with GFP. Compounds were cleared for functional testing if they exhibited a ≥70ps response for 2CS (horizontal line) and ≤50ps response for the null biosensor (vertical line). **(B)** The distribution of the 18 compounds tested in this paper after meeting the inclusion criteria in (A) is shown in green circles.

**Chemical Structures of selected hit compounds derived from the HTS FRET screening.**
FRET HTS of the DIVERset (ChemBridge, San Diego, CA) 50,000 compound library was used to detect structural changes in the human cardiac two-color SERCA2a (2CS). Application of several analysis steps, outlined in the screening tree (shown in Fig. 1B), on the fluorescence lifetimes and emission spectra in the presence of the compounds compared to that in the absence of compounds (Fig. 2 and 3) resulted in 18 hit compounds (Supplementary Fig. S1 and S2, Table S1).

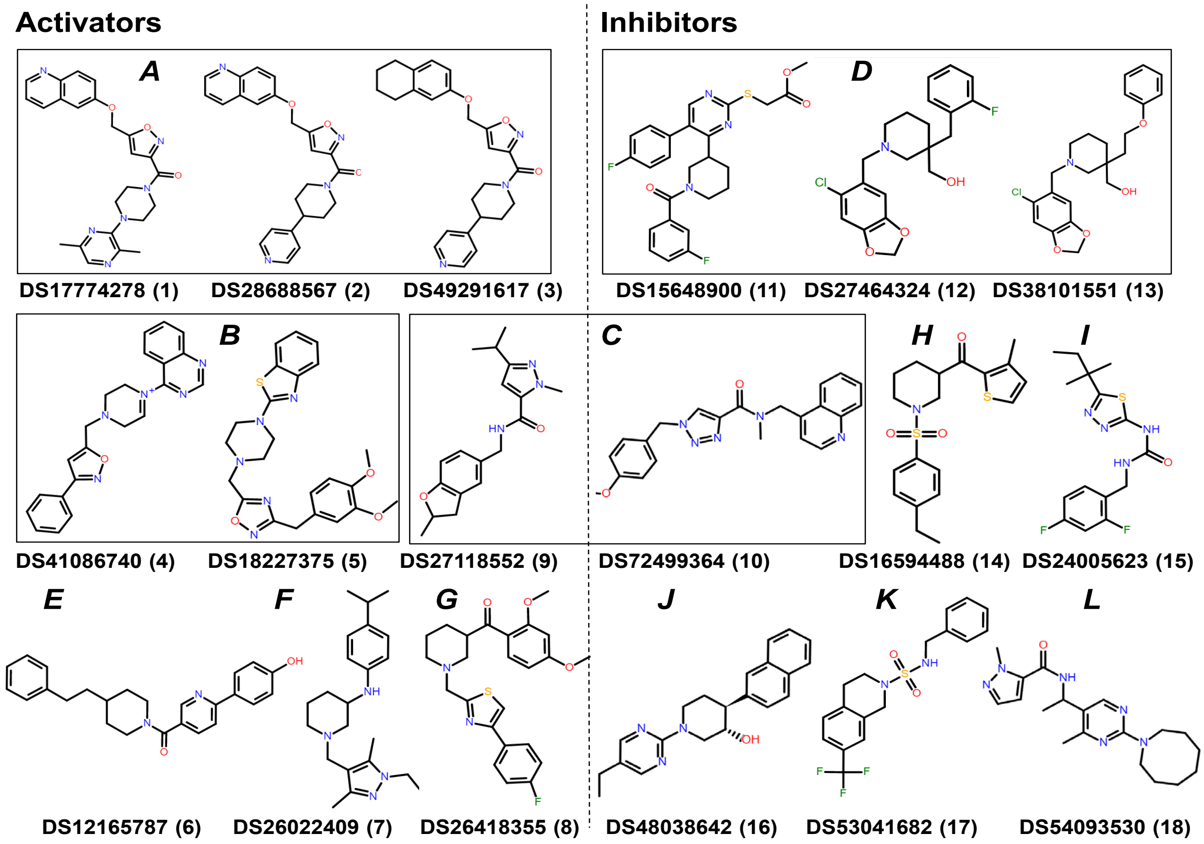


**Supplementary Figure S2.** Chemical structures of 18 hit compounds that decrease FRET *E* of 2CS by 3SD in HTS assay. The compound DIVERSet identification number (ID) and assigned numeric code (**1-18**) are listed below each compound. Compounds with a Tanimoto coefficient [1] and maximum common substructure (MCS) score above 0.4 were binned in clusters in boxes (**A-D**) and as singleton structures (**E-L**).

**Supplementary Table S1: Chemical names and physicochemical properties of hit compounds**. The compound ID from the DIVERSet 50K compound library (ChemBridge, San Diego, CA) and assigned numeric code (**1-18**) are in the first column. Compounds with a Tanimoto coefficient and maximum common substructure (MCS)[1] scores above 0.4 were binned as clusters (**A-D**), while those with scores below 0.4 were classed as singletons (**E-L**). The MSC or scaffold of the compound is in bolded text in its chemical name in 2nd column. The Lipinski's Rule of Five[2] physiochemical properties (MW, cLogP, rotatable bonds, hydrogen donors and acceptors, and total polar surface area) are listed for each compound.

| *Compound ID | Chemical Name | MW | cLogP | Rot. bonds | Hdon | HAcc | tPSA (Å) |
|---|---|---|---|---|---|---|---|
| | | **Activators** | | | | | |
| | | **A** | | | | | |
| DS17774278 (**1**) | 6-[(3-{[4-(3,6-dimethyl-2-pyrazinyl)-1-**piperazinyl]carbonyl}-5-isoxazolyl**)methoxy]quinoline | 444.5 | 3.17 | 5 | 0 | 7 | 97.5 |
| DS28688567 (**2**) | 6-[(3-{[4-(4-pyridinyl)-1-**piperidinyl]carbonyl}-5-isoxazoly**l)methoxy]quinoline | 414.5 | 4.15 | 5 | 0 | 6 | 81.4 |
| DS49291617 (**3**) | 4-[1-({5-[(5,6,7,8-tetrahydro-2-naphthalenyloxy)methyl]-3-**isoxazoly**l}**carbonyl)-4-piperidinyl**]pyridine | 417.5 | 4.5 | 5 | 0 | 0 | 68.4 |
| | | **B** | | | | | |
| DS41086740 (**4**) | 4-{4-[(3-phenyl-**5-isoxazolyl)methyl]-1-piperazinyl**}quinazoline | 371.4 | 3.6 | 4 | 0 | 5 | 58.3 |
| DS18227375 (**5**) | 2-(4-{[3-(3,4-dimethoxybenzyl)-**1,2,4-oxadiazol-5-yl]methyl**}**-1-piperazinyl**)-1,3-benzothiazole | 451.5 | 3.6 | 5 | 0 | 7 | 105 |
| | | **E** | | | | | |
| DS12165787 (**6**) | 4-(5-{[4-(2-phenylethyl)**piperidin-1-yl]carbonyl}pyridin-2-yl**)phenol | 386.5 | 4.88 | 5 | 1 | 3 | 53.4 |
| | | **F** | | | | | |
| DS26022409 (**7**) | 1-[(1-ethyl-3,5-dimethyl-1*H*-pyrazol-4-yl)methyl]-*N*-(4-isopropylphenyl)-**3-piperidinamine** | 354.5 | 4.73 | 4 | 1 | 3 | 33.1 |
| | | **G** | | | | | |
| DS26418355 (**8**) | (2,4-dimethoxyphenyl)(1-{[4-(4-fluorophenyl)-**1,3-thiazol-2-** | 440.5 | 4.37 | 7 | 6 | 0 | 79.9 |

| | | | | | | | |
|---|---|---|---|---|---|---|---|
| | **yl]methyl}-3-piperidinyl)** methanone | | | | | | |
| | | **C** | | | | | |
| DS27118552 (**9**) | 3-isopropyl-1-methyl-*N*-[(2-methyl-2,3-dihydro-1-benzofuran-5-yl)methyl]-1*H*-**pyrazole-5-carboxamide** | 313.4 | 3.19 | 4 | 1 | 3 | 56.2 |
| | | **Inhibitors** | | | | | |
| | | **C** | | | | | |
| DS72499364 (**10**) | 1-(4-methoxybenzyl)-*N*-methyl-*N*-(4-quinolinylmethyl)-1*H*-**1,2,3-triazole-4-carboxamide** | 387.4 | 3.16 | 6 | 0 | 5 | 73.1 |
| | | **D** | | | | | |
| DS15648900 (**11**) | methyl {[4-[1-(3-fluorobenzoyl)-**3-piperidinyl**]-5-(4-fluorophenyl)-2-pyrimidinyl]thio}acetate | 483.5 | 4.64 | 6 | 0 | 5 | 97.7 |
| DS27464324 (**12**) | [1-[(6-chloro-1,3-benzodioxol-5-yl)methyl]-3-(2-fluorobenzyl)-**3-piperidinyl**]methanol | 391.9 | 3.96 | 5 | 1 | 4 | 41.9 |
| DS38101551 (**13**) | [1-[(6-chloro-1,3-benzodioxol-5-yl)methyl]-3-(2-phenoxyethyl)-**3-piperidinyl**]methanol | 403.9 | 4.10 | 7 | 1 | 5 | 51.2 |
| | | **H** | | | | | |
| DS16594488 (**14**) | {1-[(4-ethylphenyl)**sulfonyl]-3-piperidinyl**}(3-methyl-2-thienyl)methanone | 377.5 | 4.92 | 3 | 0 | 4 | 91.1 |
| | | **I** | | | | | |
| DS24005623 (**15**) | *N*-(2,4-difluorobenzyl)-*N'*-[5-(1,1-dimethylpropyl)-1,3,4-**thiadiazol-2-yl]urea** | 340.4 | 4.29 | 5 | 2 | 3 | 95.2 |
| | | **J** | | | | | |
| DS48038642 (**16**) | (3S*,4S*)-1-(5-ethylpyrimidin-2-yl)-4-(2-naphthyl)**piperidin-3-ol** | 333.4 | 3.61 | 3 | 1 | 3 | 49.3 |
| | | **K** | | | | | |
| DS53041682 (**17**) | *N*-benzyl-7-(trifluoromethyl)-3,4-dihydroisoquinoline-2(1*H*)-**sulfonamide** | 370.4 | 4.50 | 2 | 1 | 2 | 57.8 |
| | | **L** | | | | | |

| | | | | | | | |
|---|---|---|---|---|---|---|---|
| DS54093530 (**18**) | *N*-{1-[2-(1-azocanyl)-**4-methyl-5-pyrimidinyl**]**ethyl**}-1-methyl-1*H*-pyrazole-5-carboxamide | 356.5 | 3.24 | 4 | 1 | 5 | 75.9 |

*DIVERSet Compound ID and Code
MW: Molecular Weight (g/mol)
clogP: calculated partition coefficient for lipophilicity: the affinity of the compound to a lipophilic environment
Rot. Bonds: non-H Rotatable bonds
Hdon: Hydrogen donor
HAcc: Hydrogen Acceptor
tPSA: total polar surface area

## Supplementary References

1 Willett, P. The Calculation of Molecular Structural Similarity: Principles and Practice. *Mol Inform* **33**, 403-413, doi:10.1002/minf.201400024 (2014).

2 Lipinski, C. A., Lombardo, F., Dominy, B. W. & Feeney, P. J. Experimental and computational approaches to estimate solubility and permeability in drug discovery and development settings. *Adv Drug Deliv Rev* **46**, 3-26, doi:10.1016/s0169-409x(00)00129-0 (2001).
